# Delineation of the Human Germinal Centre Immune Landscape Using Multiplex Imaging Analysis

**DOI:** 10.1111/imm.13955

**Published:** 2025-05-27

**Authors:** Spiros Georgakis, Michail Orfanakis, Craig Fenwick, Cloe Brenna, Simon Burgermeister, Helen Lindsay, Giuliana Xavier de Medeiros, Fernanda Romano Bruno, Susan Pereira Ribeiro, Raphael Gottardo, Giuseppe Pantaleo, Constantinos Petrovas

**Affiliations:** ^1^ Department of Laboratory Medicine and Pathology Institute of Pathology, Lausanne University Hospital and Lausanne University Lausanne Switzerland; ^2^ Service of Immunology and Allergy, Department of Medicine Lausanne University Hospital and Lausanne University Lausanne Switzerland; ^3^ Biomedical Data Science Center Lausanne University Hospital and Lausanne University Lausanne Switzerland; ^4^ Pathology Advanced Translational Research Unit (PATRU), Department of Pathology and Laboratory Medicine Emory University School of Medicine Atlanta Georgia USA; ^5^ Emory Vaccine Center Atlanta Georgia USA; ^6^ Winship Cancer Institute of Emory University Atlanta Georgia USA

**Keywords:** follicles, immune landscaping, multiplex imaging, T_FH_ cells

## Abstract

Given the role of follicular immune dynamics, especially the germinal centre, for the development of pathogen‐specific antibodies, their in situ characterisation is of great importance. We have developed a multiplex immunofluorescence imaging pipeline that allows the analysis of human follicular adaptive and innate immune cell subsets. Our data revealed the in situ phenotypic heterogeneity and differential localisation of follicular helper CD4 T (T_FH_) cell subsets across follicular areas in tonsils and reactive lymph nodes (LNs). Cell clustering analysis identified specific T_FH_ subsets with differential prevalence between tonsils and LNs. Further, a multiplex RNAscope/protein imaging assay revealed the functional heterogeneity of T_FH_ cells. No significant differences in follicular innate immune cell densities were found between tonsils and LNs. In conclusion, we present a combinatory experimental approach that provides a comprehensive analysis of human follicular and/or germinal centre immune dynamics and could be used to further understand the pathogenesis of diseases such as HIV and lymphomas.

## Graphical abstract

Human CD4 follicular helper T cells (T_FH_) are a heterogeneous cell population at the phenotypic, localisation and in situ functional level. The application of complementary methods is a prerequisite for a comprehensive delineation of the immune landscape at the tissue level.

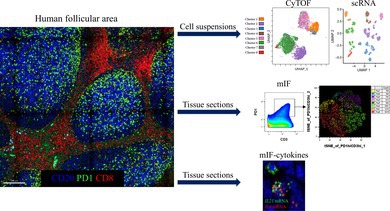

## 1 Introduction

Follicles, and more specifically germinal centres (GCs), are the lymph node (LN) anatomical areas where the development of antigen‐specific B cell responses takes place [1]. Under physiological conditions, GCs are well‐structured areas where the interaction between specific stromal and immune cells is compartmentalised to facilitate the optimal development of high‐affinity B cell responses [2]. T_FH_ cells, a critical provider of the necessary help for GC B cell proliferation and maturation [3], can interact with GC B cells through surface receptors like PD1, ICOS [3] as well as soluble mediators such as IL‐21 and IL‐4 cytokines [4]. Chronic inflammatory diseases like HIV infection [5] or angioimmunoblastic T cell lymphoma [6] have a dramatic effect on the architecture and cellularity of LNs and particularly of follicles. Therefore, the comprehensive analysis of the follicular and GC immune dynamics is of great importance to understand their role in antibody responses in natural infection or vaccination, as well as for relevant disease pathogenesis.

The differentiation of human T_FH_ cells is a multistep process characterised by the generation of cell subsets with distinct phenotypes, functions and molecular profiles [7, 8, 9]. In line with this, human follicular CD8 T cells (fCD8) represent a cell subset with distinct LN localisation and molecular profile [10]. Several studies have been focused on the analysis of LN cells using scRNA [11, 12] and spatial transcriptomic [13] methodologies. However, the comprehensive understanding of the follicular microenvironment requires complementary approaches allowing for the investigation of (i) relevant cell subsets both at mRNA and protein level (phenotypic and functional characterisation) and (ii) their spatial organisation as identified by their distribution as well as the ‘neighbouring’, or surrounding cell profiling which could provide insights for potential local immune interactions. Investigating the possible association between phenotype, function and localisation of relevant cell types [8] will help to delineate the complex cellular and molecular follicular network.

We have developed and applied a multiplex immunofluorescence (mIF) imaging approach that allows simultaneous in situ phenotypic characterisation and distribution profile of T_FH_ and fCD8 T cells in human tonsils and reactive LNs, supplemented by in situ functional characterisation of T_FH_ cells. Our analysis reveals the in situ heterogeneity of follicular T cell subsets and points to specific subsets differentially expressed between tonsils and LNs.

## 2 Methods

### 2.1 Human Material and Study Approval

The tissue samples used in this study were obtained from (i) the archives of the Institute of Pathology of Lausanne University Hospital, Switzerland (cancer‐free, HIV‐free reactive LNs) and their use was approved by the Ethical Committee of the Canton de Vaud, Switzerland (2021‐01161) and (ii) from the Hospital de l'Enfance of Lausanne (tonsils were obtained from anonymised children during routine tonsillectomy) and their use was approved by the Canton de Vaud‐CER‐VD, Switzerland (PB_2016‐02436 (201/11)). Tissues from participants with written consent were used, while all procedures were in accordance with the Declaration of Helsinki.

### 2.2 Cell Phenotyping With Mass Cytometry (CyTOF)

Part of the tonsillar tissues was dedicated to preparing a single‐cell suspension. Briefly, after removing fatty and ‘bloody’ parts, the tissue was cut into small pieces and single cells were extracted by mechanical disruption and filtering through cell strainers. Cell preparations were kept in liquid nitrogen until further use. Cryopreserved tonsil mononuclear cells (TNMCs) were thawed and resuspended in complete RPMI medium (Gibco; Life Technologies; 10% heat‐inactivated FBS [Institut de Biotechnologies Jacques Boy], 100 IU/mL penicillin and 100 μg/mL streptomycin [BioConcept]). Before staining, cells were rested for 6 h at 37°C with 5 U/mL Benzonase (Thermo Fisher) to prevent cell clumping. Cells were washed in PBS containing 0.5% BSA (Sigma) and incubated for 20 min with a 50 μL antibody cocktail of cell surface metal conjugated antibodies (Table 1). Cell viability staining was performed using the Cell‐IDTM‐103 Rh Intercalator at a final concentration of 1 μM. Cells were washed and fixed for 10 min at RT with 2.4% paraformaldehyde (PFA; Thermo Fisher) in PBS. Next, cells were permeabilised for 45 min at 4°C with Foxp3 Fixation/Permeabilisation Kit (eBioscience), washed and stained at 4°C for 30 min with a 50 μL cocktail of intracellular metal conjugated antibodies (Table 2). For TCF1 assessment, the antibody is conjugated with phycoerythrin fluorochrome (Clone 7F11A10, Cat. No. 655208, BioLegend) and then detected by metal conjugated anti‐PE (145Nd, Standard Biotools). Cells were washed and fixed for 10 min at RT with 2.4% PFA. Total cells were identified by DNA intercalation (1 mM Cell‐ID Intercalator, Standard Biotools) in 1% PFA and 0.3% saponin (Sigma) at 4°C overnight. For CyTOF analysis, cells were washed three times with MilliQ water and resuspended at 0.5 × 10^6^ cells/mL in 0.1% EQ Four Element Calibration Beads Solution (Standard Biotools). Labelled samples were assessed by using a Helios Mass Cytometer Instrument (Standard Biotools), using a flow rate of 0.030 mL/min and an event rate of ~300 cells/s. Flow cytometry standard (FCS) files were normalised to EQ Four Element calibration beads using CyTOF software. For conventional cytometric analysis of immune cell populations, FCS files were imported into Cytobank data analysis software for processing, with more in‐depth analysis performed in R using the OpenCyto and cytofkit packages.

**TABLE 1:** Antibodies used for the mass cytometry phenotypic characterisation of tonsillar‐derived cells.

Antibody	Metal	Clone	Company	Cat. No.	Amount
Rhodium	103Rh	Cell‐ID	Standard Biotools	201103A	0.6 μL
CD8	113ln	RPA‐T8	BioLegend	301018	0.6 μL
CD4	115In	RPA‐T4	BioLegend	300515	0.6 μL
CCR6	141Pr	11A9	Standard Biotools	3141014A	1 μL
CD19	142Nd	HIB19	Standard Biotools	3142001B	1.6 μL
CCR6	144Nd	G034E3	Standard Biotools	3141003A	1.88 μL
CD20	147Sm	2H7	Standard Biotools	3147001B	0.7 μL
ICOS	148Nd	C398.4A	Standard Biotools	3148019B	1 μL
CCR4	149Sm	L291H4	Standard Biotools	3149029A	1.5 μL
CD40L	152Sm	TRAP1	BD Biosciences	555698	3 μL
TIGIT	153Eu	MBSA43	Standard Biotools	3153019B	2 μL
CD3	154Sm	UCHT1	Standard Biotools	3154003B	0.5 μL
CD27	155Gd	L128	Standard Biotools	3155001B	1 μL
CXCR3	156Gd	G025H7	Standard Biotools	3156004B	0.8 μL
CCR7	159 Tb	G043H7	Standard Biotools	3159003A	1 μL
CD30	162Dy	BY88	BioLegend	333902	3 μL
CXCR5	164Dy	RF8B2	Standard Biotools	3164029B	1.2 μL
CD45RO	165Ho	UCHL1	Standard Biotools	3165011B	0.7 μL
CD38	167Er	HIT2	Standard Biotools	3167001B	0.6 μL
CD45RA	170Er	HI100	Standard Biotools	3171002B	1.6 μL
HLA‐DR	174Yb	L243	Standard Biotools	3174001B	0.7 μL
CD279/PD‐1	175Lu	EH12.2H7	Standard Biotools	3175008B	1 μL
CD127	176Yb	A019D5	Standard Biotools	3176004B	0.7 μL
CTLA4	194Pt	Ipilimumab	BMS	ACB3065	2.5 μL
CD57	198Pt	NK‐1	BD Biosciences	555618	1 μL
CD16	209Bi	3G8	Standard Biotools	3209002B	1.5 μL

**TABLE 2:** Antibodies used for the mass cytometry intracellular characterisation of tonsillar‐derived cells.

Antibody	Metal	Clone	Company	Cat. No.	Amount
Anti‐PE	145Nd	PE001	Standard Biotools	3145006B	2 μL
GATA‐3	146Nd	TWAJ	ThermoFisher	14‐9966‐82	1 μL
Tbet	161Dy	4B10	Standard Biotools	3161014B	2 μL
Bcl6	163Dy	K112‐91	Standard Biotools	3163012B	2 μL
Ki67	168Tm	Ki‐67	Standard Biotools	3168001B	1.4 μL
Blimp1	169Tm	646702	Bio‐Techne	MAB36081	3 μL
Granzyme B	171Yb	GB11	Standard Biotools	3171002B	2 μL
Bcl2	173Yb	100	BioLegend	658702	2 μL

### 2.3 Flow Cytometry

Cryopreserved tonsillar cell suspensions were thawed and stained with anti‐human mAbs (10^6^ cells per test). Surface staining was performed at room temperature (RT) for 20 min. Samples underwent fixation with the fix buffer from BD Transcription Factor Buffer Set (Cat. No. 562574) (4.2% PFA) for 15 min at RT. Cells were then washed with stain buffer and resuspended in 200 μL for acquisition. Acquisition was performed on an A5 Symphony flow cytometer (BD Biosciences) driven by BD FACSDiva software. Acquired data were analysed using FlowJo v.10.8.1. LIVE/DEAD Fixable Aqua Dead Cell Stain was used to gate on live cells. Cells were phenotyped using the panel in Table 3.

**TABLE 3:** The clones, fluorochromes used for their detection and the catalogue numbers for the flow cytometry antibodies used are shown.

Fluorocrome	Antibody	Clone	Lot	Cat. #	Brand
BV510	Live/Dead	—	2729807	L34966	Invitrogen
BUV805	CD3	UCHT1	73290	612895	BD Horizon
AF700	CD4	L200	5345947	557922	BD Pharmingen
BUV496	CD8	RPA‐T8	1067799	612942	BD Horizon
BV570	CD19	HIB19	B307540	302 235	BioLegend
PE	CD14	M5E2	4078214	561707	BD Pharmingen
BUV661	CD16	3G8	1039771	750284	BD OptiBuild
BUV615	CD11c	3.9	191038	624297	BD Horizon
PE‐CF594	CD123	7G3	1041160	562391	BD Horizon
BV650	CD11b	ICRF44	1039762	740566	BD OptiBuild
BUV563	CD15	W6D3	1039769	741417	BD OptiBuild
BV421	CD66b	G10F5	B232289	305112	BioLegend
BV786	PD1	EH12.2H7	B410666	329930	BioLegend
BUV395	ICOS	DX‐29	3233604	564777	BD Horizon
BV750	CXCR5	RF8B2	1124740	747111	BD OptiBuild
FITC	CD163	GHI/61	9023965	563697	BD Pharmingen

### 2.4 Tonsilar scRNA Analysis

We examined single‐cell RNA‐seq expression for markers of interest using the CD4 T‐cell data provided in the R package HCATonsilData (version 1.2) [14]. To avoid contamination by doublets or miss‐annotated cells, we removed cells with more than one CD20, CD8A, CD8B or CD8B2 read, or with counts of any of these higher than the count for CD4. We further selected cells with at least two reads from PD‐1/PDCD1. For consistency with the imaging analyses, we use gating terminology to describe the selected cells, though note that CD4 T‐cells were defined in the original publication by clustering, not gating. We performed clustering and Uniform Manifold Approximation and Projection (UMAP) analyses on the selected cells from five child donors processed at the same hospital using the expression of PDCD1(PD1), CXCR5, CXCR3, B3GAT1(CD57), TIGIT, ICOS, BCL6, MKI67, GATA3, TCF7 and GZMB. Analyses were performed using R version 4.4.2 [15] and packages scater v.1.32.1 (https://www.r‐project.org/) [14], scran v.1.32.0 [16] and Summarised Experiment v.1.34.0 (https://www.bioconductor.org/packages/release/bioc/html/SummarizedExperiment.html#:~:text=URL‐,https%3A//bioconductor.org/packages/SummarizedExperiment,‐Bug%20Reports).

### 2.5 Tissue Processing and Staining

Fresh tissues were fixed in formalin overnight as soon as possible after biopsy and processed for the preparation of formalin‐fixed, paraffin‐embedded (FFPE) blocks using standard procedures. The blocks were sequentially cut into 4–5 μm sections and prepared on Superfrost glass slides (Thermo Scientific, Waltham, MA, USA, Ref. J1800AMNZ), dried overnight and stored at 4°C. Before staining, the slides were heated on a metal hotplate (Stretching Table, Medite, Burgdorf, OTS 40.2025, Ref. 9064740715) at 65°C for 30 min. Tissue sections were stained with titrated antibodies (Table 4) using a Ventana Discovery Ultra Autostainer (Roche Diagnostics, Ventana Medical Systems, Tucson, AZ, 85755, USA). Tissues were deparaffinised and hydrated, and the protein epitopes were retrieved by applying the standard Ventana Discovery protocols. Before all antibody incubation steps, tissues were blocked using Antibody Diluent/Block from Akoya (ARD1001EA, Akoya Biosciences, Marlborough, MA 01752, USA). The cycling staining/imaging approach that we developed consists of two staining cycles (Table 4, T and innate panel). During the first staining cycle, unconjugated and conjugated primary antibodies coupled with Alexa Fluor dyes were used. Both unconjugated and conjugated antibodies were diluted in Antibody Diluent/Block and incubated sequentially for 90 min at RT. Alexa Fluor‐conjugated antibodies were diluted in Antibody Diluent/Block and incubated for 45 min at RT. To avoid any unwanted secondary antibody binding to conjugated antibodies, we started by incubating unconjugated antibodies, followed by secondary antibodies and lastly with the conjugated antibodies. The samples were then counterstained with SYTO45 (1/10 000 dilution in TBS‐T, Cat. No. 10297192, ThermoFisher Scientific for 40 min), rinsed in soapy water and mounted using DAKO mounting medium (Dako/Agilent, Santa Clara, CA, USA, Ref. S302380‐2). After imaging the first cycle, slide coverslips were carefully de‐mounted using warm ddH_2_O, and slides were washed briefly in PBS. Then, the first cycle's antibodies were stripped off using Cell Conditioning Solution (CC2, 950‐223, Roche Diagnostics) for 10 min at 100°C. After the stripping step, slides were washed 2× (~10 s) in ddH_2_O and 1× for 5 min in PBS, and the second cycle of staining followed. During the second cycle, Opal dyes (Opal 7‐colour Automation IHC kit, from Akoya, Ref. NEL821001KT and Opal650 reagent pack FP1496001KT) were used to amplify the signal of the second cycle primary antibodies (Table 4). More specifically, tissue sections were sequentially subjected to antibody blocking, staining with primary antibodies, incubation with secondary HRP‐conjugated antibodies (DISCOVERY OmniMap anti‐Ms HRP/760‐4310, DISCOVERY OmniMap anti‐Rb HRP/760‐4311) for 16 min, detection with optimised fluorescent Opal tyramide signal amplification (TSA) dyes and repeated antibody denaturation cycles. The samples were then counterstained with SYTO40 (1/10 000 dilution in TBS‐T, Cat. No., ThermoFisher Scientific for 40 min), rinsed in soapy water and mounted using DAKO mounting medium (Dako/Agilent, Santa Clara, CA, USA, Ref. S302380‐2). If needed, at the end of the second staining cycle, slides were incubated with CD57‐BV421 antibody for 90 min, RT as an additional registration marker facilitating image alignment.

**TABLE 4:** The antibody clones, fluorochromes used for their detection as well as the corresponding staining panels used are shown.

Epitope	Ab Clone	Fluorophore	Conjugated/unconjugated	Cat. No.	Panel	Cycle
CD3	OTI3E10	Alexa‐546	Unconjugated	TA506064	T cell	1
CD4	Polyclonal	Alexa‐700	Conjugated	FAB8165N‐100	T cell Treg	1
CD20	L26	eF615	Conjugated	42‐0202‐82	T cell	1
CD8	C8/144B	Alexa‐568	Unconjugated	M710301‐2	T cell	1
Ki67	B56	V450	Conjugated	561281	T cell	1
PD‐1	Polyclonal	Alexa‐488	Conjugated	FAB7115G	T cell	1
CD57	QA17A04	BV421	Conjugated	393326	T cell	1, 2
TIGIT	BLR047F	Opal‐620	Unconjugated	AB243903	T cell	2
TCF‐1	C63D9	Opal‐570	Unconjugated	2203S	T cell	2
ICOS	SP98	Opal‐480	Unconjugated	AB105227	T cell	2
GRZb	GrB‐7	Opal‐650	Unconjugated	MON7029C	T cell	2
BCL6	GI191E/A8	Opal‐690	Unconjugated	760‐4241	T cell	2
CXCR‐3	1C6	Opal‐780	Unconjugated	557183	T cell	2
GATA‐3	L50‐823	Opal‐520	Unconjugated	CM405B	T cell	2
CD20	L26	Opal‐520	Unconjugated	NCL‐L‐CD20‐L26	Innate	2
CD31	WM59	Alexa‐594	Conjugated	303126	Innate	1
IL‐21	Polyclonal	BV421	Unconjugated	AHP1845	Innate	1
IL‐10	E10	Opal‐620	Unconjugated	SC‐8438	Innate	2
FDC	CNA.42	Alexa‐488	Unconjugated	14‐9968‐82	Innate	1
CD68	KP‐1	Alexa‐790	Conjugated	sc‐20 060 AF790	Innate	1
CD163	EDHu‐1	Alexa‐647	Conjugated	NB110‐40686AF64	Innate	1
CD11c	EP1347Y	Alexa‐555	Conjugated	ab279329	Innate	1
MPO	Polyclonal	Opal‐690	Unconjugated	AB9535	Innate	2
CD123	BSB‐59	Opal‐570	Unconjugated	BSB5326	Innate	2
CXCL‐13	Polyclonal	Opal‐780	Unconjugated	PA528827	Innate	2
Mouse IgG2b	Polyclonal	Alexa‐546	Conjugated	A‐21143	T cell	1
Mouse IgG1	Polyclonal	Alexa‐568	Conjugated	A‐21124	T cell	1
Mouse IgM	Polyclonal	Alexa‐488	Conjugated	115‐547‐020	Innate	1
Rabbit IgG	Polyclonal	BV421	Conjugated	565014	Innate	1
FOXP3	SP97	Alexa‐594	Conjugated	AB275080	Treg	1
CD20	L26	Alexa‐546	Unconjugated	NCL‐L‐CD20‐L26	Treg	1
CD25	4C9	Alexa‐488	Unconjugated	BSB6321	Treg	1
PD1	NAT105	Opal‐650	Unconjugated	ACI3137CK	Treg	1
Mouse IgG2b	Polyclonal	Alexa‐488	Conjugated	A21141	Treg	1
Mouse IgG2a	Polyclonal	Alexa‐546	Conjugated	A‐21133	Treg	1

### 2.6 Multiplex RNAscope

RNAscope in situ hybridisation for IL21, IL4 and IFNγ RNA visualisation was performed according to the manufacturer's instructions using the RNA probes (Hs‐IL21, Cat. No. 401251‐C1, Hs‐IL4, Cat. No. 315191‐C2, Hs‐IFNg, Cat No. 310501‐C3, Advanced Cell Diagnostics, Hayward, CA, USA) and the RNAscope Multiplex Fluorescent Reagent Kit v2 (Advanced Cell Diagnostics) with small modifications. Tonsillar samples were deparaffinised by applying the standard Ventana Discovery's protocols. Then, we treated the sections with RNAscope Hydrogen Peroxide for 10 min at RT, followed by an antigen retrieval step at 100°C for 15 min. Subsequently, sections were incubated with Protease III for 15 min at 40°C in a HybEz hybridisation oven (ACD). Sections were incubated with the *IL21*, *IL4* and *IFNG* specific probes at 40°C for 2 h, and then proceeded with three signal amplification cycles using the RNAscope amplification reagents. To visualise the RNA signals, we used a tyramide‐based detection system by Akoya as mentioned above (Opal 7‐colour Automation IHC Kit, from Akoya, Ref. NEL821001KT and Opal540 reagent pack FP1494001KT). After the RNAscope protocol, we subjected the tissue sections to antibody blocking for 30 min, and then we proceeded with the protein marker staining (Table 5). Applied antibodies were verified for their compatibility with the RNAscope procedure. Both unconjugated and conjugated antibodies were diluted in Antibody Diluent/Blocker and incubated for 90 min RT. Alexa Fluor conjugated secondary antibodies were diluted in Antibody Diluent/Blocker and incubated for 45 min RT. The samples were then counterstained with DAPI and mounted using DAKO mounting medium.

**TABLE 5:** The antibody clones, RNA probes and fluorochromes used for the Multiplex RNAScope panel.

Epitope	Ab Clone	Fluorophore	Conjugated/unconjugated	Cat. No.
CD3	OTI3E10	Alexa‐633	Unconjugated	TA506064
PD‐1	Polyclonal	Alexa‐488	Conjugated	FAB7115G
CD57	QA17A04	Alexa‐BV421	Conjugated	393326
CD20	L26	Alexa‐546	Unconjugated	NCL‐L‐CD20‐L26
Mouse IgG2b	Polyclonal	Alexa‐633	Conjugated	A‐21146
Mouse IgG2a	Polyclonal	Alexa‐546	Conjugated	A‐21133
Hs‐IL21	C1	Opal‐620	Unconjugated	401251‐C1
Hs‐IL4	C2	Opal‐540	Unconjugated	315191‐C2
Hs‐IFNg	C3	Opal‐690	Unconjugated	310501‐C3

### 2.7 Data Acquisition

Images were acquired using a Leica Stellaris 8 SP8 confocal system, equipped with Leica Application Suite X (LAS‐X)‐4.6.1.27508 software, at 512 × 512‐pixel density, 0.75× optical zoom and a z‐step of 1 μm using a 20× objective (0.75 NA). For RNAscope data acquisition, a 40× (0.95 NA) and a 63× (1.4 NA) objective were used. Frame averaging or summing was never used while obtaining the images. At least 70% of each section was imaged to ensure an accurate representation and minimise selection bias. Tissues stained with a single antibody fluorophore combination were used to create a compensation matrix via the Leica LAS‐AF Channel Dye Separation module (Leica Microsystems), which was used to correct fluorophore spillover (when present), as per the user's manual. When the dye separation results were not optimal, the manual LAS‐AF Channel Dye Separation module was employed.

### 2.8 Image Alignment and Registration—Cell Segmentation

The alignment of the images generated during the two cycles was performed using SimpleITK [17] as an Imaris extension (Imaris software version 9.9.0, Biplane). To facilitate registration, we utilised one or two common channels present in both imaging cycles (SYTO or CD57‐BV421). After successful alignment, the Surface Creation module of Imaris was used to generate three‐dimensional segmented surfaces (based on the nuclear signal) of spillover‐corrected images. Segmented cells were then processed with the filtering Imaris module using different combinations of filtering types based on the mean and median intensities of channels to exclude artefacts that are characterised by a uniform staining across the segmented area. Areas with uniform staining were excluded among the different tissues. Data generated, such as average voxel intensities for all channels, in addition to the volume and sphericity of the three‐dimensional surfaces, were exported in Microsoft Excel format.

### 2.9 Quantitative Imaging Analysis (Histocytometry)

The Excel files obtained from cell segmentation were converted to comma‐separated value (.CSV) files, and data were imported into FlowJo (version 10) for further analysis. Well‐defined follicular areas were included in the analysis of follicular/GC immune landscape, and the data were quantified as relative frequencies (%), as absolute counts, or as normalised cell counts (using the DownSampleV3 plugin of FlowJo). We should mention that any background or irregular staining was limited in extrafollicular areas. Imaging data were analysed either as individual samples or by applying a batch analysis. For the analysis of individual samples, hand gating was performed for the identification of relevant cell subsets, and the intensities of individual biomarkers used in gated populations were presented as histograms or 2D contour plots. Follicular areas were identified based on the density of CD20^hi/dim^, a biomarker specific for B cells The cut‐off values for the identification of cells expressing ‘high’ profile for a given biomarker (e.g., CD3, CD4 and PD1) were determined based on the 2D plot expression profile for this biomarker on relevant cells (e.g., PD1 expression on CD3 vs. CD20 cells), using Histocytometry analysis and the inspection of its intensity in the raw mIF image. Histocytometry analysed cells of interest were exported and imported into Imaris raw mIF image as segmented spots for the comparison/validation of these cells to their original counterparts. For batching analysis, the same number was assigned to all segmented cells from a given tissue, and all raw intensities exported from Imaris were normalised. Data normalisation was performed using the StandardScaler from the sklearn library in Python 3.9.13. First, we selected the relevant columns containing the raw mean intensities of all markers for normalisation, excluding *X*/*Y* coordinates, Volume, and Sphericity columns. The selected data was scaled to have a mean of 0 and a standard deviation of 1. The normalised data was then reconstructed into a DataFrame, ensuring that excluded columns were preserved in their original form. Regions of interest (ROIs‐follicular areas) were identified for each donor, and relevant cell subsets (e.g., follicular CD3^hi^PD1^hi^, CD3^hi^CD8^hi^CD4^lo^) were manually gated. The same number of cells (2200 for T_FH_ and 3000 for CD8 T cells) was concatenated together with the associated biomarkers under investigation. The concatenated ‘sample’ was processed using FlowJo10 modules/plugins (t‐SNE, FlowSOM [3.0.18] and Cluster Explorer) and the generated subpopulations were applied to all individual tissues included in the concatenated sample and were able to be identified based on the assigned ID number. For t‐SNE analysis, the Auto (opt‐SNE) learning configuration, 1000 iterations, perplexity 30, using the KNN algorithm (Exact‐vantage point tree) settings were used. FlowSom analysis was carried out using SOM grid size of 10 × 10, minimum spanning tree (view layout), a node scale of 100%, and the number of meta clusters of 8. Heatmaps of generated subpopulations were generated using Python 3.9.13 to visualise the normalised data. To enhance the readability of the values, the data was transformed using a natural logarithmic scale (log1*p*). We then discretised the transformed values and used the seaborn library to create a heatmap with the ‘coolwar’ colour map. The heatmap displayed a colour intensity range corresponding to the markers' expression levels.

### 2.10 Cell Distribution and Distance Analysis

In situ distribution of individual cell types and the spatial relationship between GC B cell and T_FH_ cell subsets were investigated across individual ROIs, with at least 20 cells of the corresponding cell subsets, by (i) analysing the experimental curves generated from Ripley's G function and the theoretical Poisson curve using Pointpats 2.3.0 (https://doi.org/10.5281/zenodo.7706219). The area between the empirical and theoretical Poisson curve was calculated using the Numpy library [18], (ii) calculating the minimum Euclidean distance between individual GC B and T_FH_ cells using Python 3.10.9 and the Scipy library [19]. The matrix interaction was generated using *X* and *Y* coordinates from each cell type, and the median distance was extracted, and (iii) analysing the cross‐G function for the calculated minimum distances between corresponding GC B and T_FH_ cell subsets. The extracted data were presented either as bar graphs (*y*‐axis: range of all measured distances, *y*‐axis: count of B cells for any given distance) or dot plots representing the mean values of corresponding measurements in individual follicular areas.

### 2.11 Statistics

Data were analysed using the Mann–Whitney or Wilcoxon test. Graphs were generated using GraphPad Prism (8.3.0), and significance was determined at a threshold of *p* value < 0.05.

## 3 Results

### 3.1 Human T_FH_ Cells Are Phenotypically Heterogeneous Cellular Compartment

**FIGURE 1:**
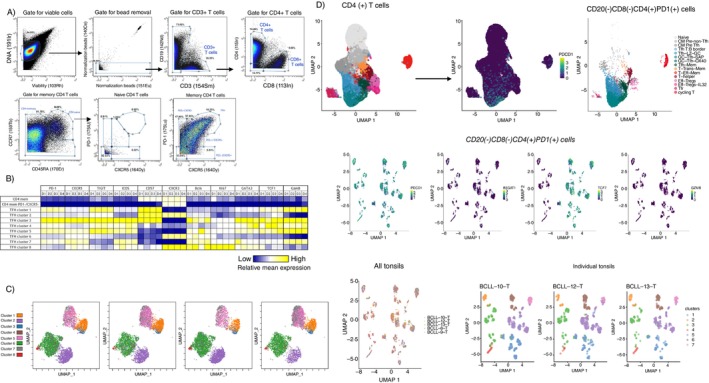
T_FH_ cells constitute a phenotypically heterogeneous cell compartment. (A) Tonsillar single cell suspensions (*n* = 4) were analysed using a CyTOF (*N* = 34 parameters) assay. The gating scheme for the identification of main cell subsets is shown. (B) Heatmap showing the relative mean expression of selected protein markers in bulk CD4^hi^ memory, CD4^hi^CXCR5^lo^PD1^lo^ memory and T_FH_ cell subsets (*n* = 8) in different tonsils (D1‐D4). A colour indicator is provided. (C) UMAP analysis demonstrating the clustering of T_FH_ clusters across four individual donors based on selected protein markers. (D) UMAP analysis showing the original clusters and annotations of CD4 tonsillar cells based on scRNA analysis (upper, left panel). The prevalence of PD1^hi^ cells (upper, middle panel) and the subset of CD8^−^CD20^−^CD4^+^PD1^+^ T_FH_ cells (upper, right panel) are shown. Expression of selected marker genes (middle row) and cluster membership per donor (lower row, right) are shown for UMAPs and re‐clustering generated using the selected cells from five donors and the genes selected from protein analyses (lower row, left).

We started our investigation by analysing tonsillar single‐cell suspensions using a multiparametric CyTOF assay (Figure 1A). Given their abundance for relevant cell subsets, tonsils represent a prototype organ for the study of follicular immune cellularity. Our initial clustering analysis revealed four phenotypically distinct T_FH_ cell subsets (Figure S1A, upper panel) in line with our previous data using human LNs [8]. Of note, a diverse expression of certain biomarkers (e.g., CD57, TIGIT, CXCR3 and Bcl2) by T_FH_ cells among the four subsets analysed was observed (Figure S1A, lower panel). More specifically, a higher expression of GATA3, TCF1 (a stemness marker for T cells [20]), and Bcl2 was observed in T_FH_ cells compared to the memory CD4 T cell compartment, whereas CD3 expression was lower in certain T_FH_ cell subsets compared to non‐T_FH_ cells, consistent with previous findings [8] (Figure S1A, lower panel). Then, we performed a clustering analysis for the CD3^hi^CXCR5^hi^PD1^hi^ cells based on the expression of markers included also in our multiplex imaging assay (see Section 2). Once again, a wide range of expression of the biomarkers used was found within the identified clusters of T_FH_ cells, as well as between the different donors (Figures 1B and S1B). The UMAP analysis further demonstrated the heterogeneity of the T_FH_ cell compartment among different donors (Figure 1C). The heterogeneity of T_FH_ cell subsets was further investigated at the gene expression level. To this end, an online public data set was used for the identification of tonsillar bulk or PD1^hi^ CD8^lo^CD20^lo^CD4^hi^ T cell subsets (Figure 1D, upper panel). Sub‐clustering of the CD8^lo^CD20^lo^CD4^hi^PD1^hi^ cells using the genes corresponding to the proteins used for the CyTOF UMAP analysis revealed a similar expression pattern between protein and corresponding genes (e.g., high prevalence of *TIGIT* and *Tcf7* in contrast to low prevalence of *B3GAT1*, a surrogate for CD57, and GrzB gene expression) (Figure 1D, middle panel and Figure S1C). Furthermore, UMAP analysis highlighted the heterogeneity of the T_FH_ cell compartment (Figure 1D, lower panel), which is in line with the heterogeneity revealed by protein analysis (Figure 1C). Our data indicate that human T_FH_ cells represent a heterogeneous cellular population with differential prevalence of specific T_FH_ subsets among individuals.

### 3.2 In Situ Phenotypic Heterogeneity T_FH_ Cells

**FIGURE 2:**
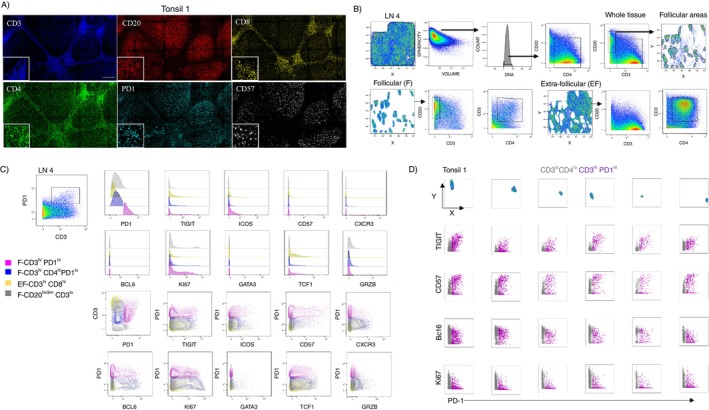
In situ phenotypic heterogeneity of T_FH_ cells revealed by mIF imaging. (A) Representative fluorescent images showing the expression of biomarkers CD3 (blue), CD20 (red), CD8 (yellow), CD4 (green), PD1 (cyan) and CD57 (grey) used in the mIF assay. Representative images from one tonsil are shown (scale bar: 150 μm). (B) The histocytometry gating scheme for the identification of B (CD20^hi/dim^), CD3^hi^, CD4^hi^ and follicular (F) CD3^hi^PD1^hi^ cells in an LN is shown. Individual follicular areas were identified based on the density of CD20^hi/dim^ cells. All follicular areas were combined (using the FlowJo10 module in one follicle for downstream analysis. The total extrafollicular area is shown. (C) The expression (raw mean fluorescence intensities) of individual biomarkers in F‐CD3^hi^PD1^hi^ T_FH_, F‐CD3^hi^CD4^hi^PD1^lo^ non‐T_FH_, extrafollicular (EF)‐CD3^hi^CD8^hi^ and F‐CD20^hi/dim^CD3^lo^ B cells are shown as histograms (upper two rows) or 2D contour plots (biomarker vs. PD1) (lower two rows). Imaging data from an LN were used. (D) Six Histocytometry identified follicular areas (top row) and 2D dot plots showing the associated expression of biomarkers used vs. PD1 in CD3^lo^CD4^lo^ (as reference cell subset‐grey) and CD3^hi^PD1^hi^ T_FH_ cells (purple).

Next, we aimed to investigate the in situ phenotypic heterogeneity of T_FH_ cells. To this end, we developed a ‘cycling’ mIF assay (membrane/cytosolic, *n* = 10) and nuclear protein (*n* = 4) markers combined with nuclear dye allowing for the comprehensive phenotypic analysis of follicular CD4^hi^CD3^hi^ T_FH_ cells (Figures 2A and S1D). The generated images were aligned, merged (Figure S1E), and the extracted coordinates were analysed with histocytometry [21, 22]. The gating scheme used for the identification of follicular areas (enriched in CD20^hi/dim^ B cells) and extrafollicular areas, as well as the follicular CD3^hi^ T cells expressing a PD1^hi^ phenotype, is shown (Figures 2B and S2A). The analysis of imaging data revealed a subset of CD3/CD4 T cells expressing also a CD20^dim^ phenotype (Figures 2B and S2A), most likely representing spillover of CD20 signal into T cells attached/engaged to B cells [23]. Interestingly, this was also a profile found when tonsillar single cell suspension was analysed with CyTOF (CD3^hi^CD19^dim^ cells) (Figure 1A) or flow cytometry (Figure S2B). Although the majority of PD1^hi^ and CD57^hi^ cells express a CD3^hi^CD4^hi^ phenotype, we observed an inconsistency with respect to the co‐expression of CD57 and CD4 in some of the analysed tissues, with a fragment of CD57^hi^ cells expressing a CD3^hi^CD4^lo^CD8^lo^PD1^hi^ phenotype (Figure S2C). To avoid underestimating the PD1^hi^CD57^hi^ T_FH_ cell subset, we identified T_FH_ cells based on the CD3^hi^PD1^hi^ phenotype in total follicular (F) areas (Figures 2B,C and S2D). Downstream analysis of individual T_FH_ cell biomarkers (e.g., ICOS, TIGIT and Bcl6) confirmed their overexpression in T_FH_ compared to non‐T_FH_ (CD3^hi^CD4^hi^PD1^lo^) and CD3^hi^CD8^hi^ cells (Figures 2C and S2D). Furthermore, our analysis allows for the back‐gating of digitally identified cells with a given phenotype and compares them to their originally, mIF‐identified counterparts (Figure S2E). Analysis of the corresponding biomarkers in CD3^hi^PD1^hi^ T_FH_ cell compartment showed a diverse expression of individual T_FH_ cell subsets in different follicles across a tissue section (Figures 2D and S3A). Altogether, our experimental approach enables the characterisation of in situ phenotypic heterogeneity of human T_FH_ cells by assessing the expression of relevant biomarkers both at the global tissue level and within individual follicular regions.

### 3.3 Distinct Spatial Organisation of Specific T_FH_ Cell Subsets

**FIGURE 3:**
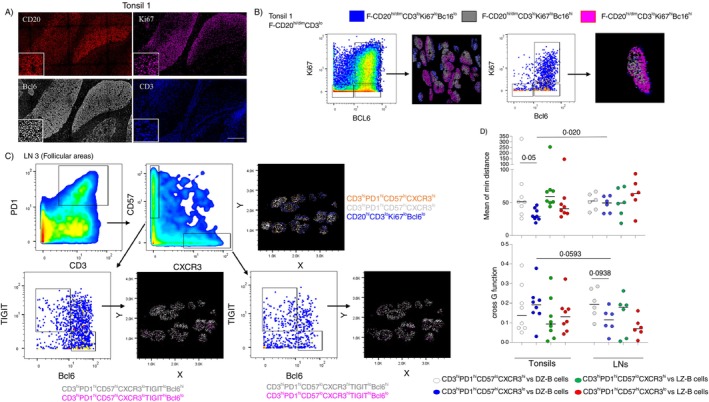
Differential localisation and distribution profile among T_FH_ cell subsets. (A) Representative fluorescent images showing the expression of CD20 (red), Ki67 (magenta) and Bcl6 (grey) in tonsillar follicles (scale bar: 300 μm). (B) 2D plots showing the expression of Ki67 vs. Bcl6 in total follicular (F) area from one tonsil and the corresponding localisation of F‐CD20^hi/dim^CD3^lo^Ki67^lo^BCl6^lo^ (blue‐MZ), F‐CD20^hi/dim^CD3^lo^Ki67^hi^BCl6^hi^ (magenta‐DZ) and F‐CD20^hi/dim^CD3^lo^Ki67^lo^BCl6^hi^ (grey‐LZ) (left). The relevant B cell subsets and their localisation for one representative follicle are also shown (right). (C) 2D plots showing the gating for the identification of B subsets (based on the expression of Ki67 and Bcl6) and CD3^hi^PD1^hi^ T_FH_ cell subsets (based on the expression of CD57, CXCR3, TIGIT and Bcl6) as well as their localisation across the areas of individual follicles (data from one LN). (D) Dot plot graphs showing accumulated data for the analysis of the minimum distance between DZ (CD20^hi/dim^CD3^lo^Ki67^hi^Bcl6^hi^) (open and blue circles) or LZ (CD20^hi/dim^CD3^lo^Ki67^lo^Bcl6^hi^) (green and red circles) B cells and T_FH_ cell subsets (CD3^hi^PD1^hi^CD57^lo^CXCR3^hi^, CD3^hi^PD1^hi^CD57^hi^CXCR3^lo^) (upper panel) and the corresponding calculated cross‐G function (lower panel). Follicular areas from two tonsils and two LNs were analysed, each dot represents a follicle. Statistical analysis was performed using the Wilcoxon and Mann–Whitney tests, and *p* values are listed.

The inclusion of Ki67 and Bcl6 biomarkers in our staining panel (Figure 3A) allows for the analysis of follicular and GC‐B cell subsets and their localisation across follicular areas (Figures 3B and S3B). We detected a distinct localisation pattern between CD20^hi/dim^CD3^lo^Ki67^lo^Bcl6^lo^ (outlet zone of the follicle‐mantle zone), proliferating CD20^hi/dim^CD3^lo^Ki67^hi^Bcl6^hi^ (centroblasts, mainly found in the Dark Zone‐DZ) and non‐proliferating CD20^hi/dim^CD3^lo^Ki67^lo^Bcl6^hi^ (mainly found in the Light Zone‐LZ) cells (Figures 3B and S3B).

Next, the localisation of specific T_FH_ cell subsets was investigated. CD57 expression is associated with highly differentiated T_FH_ cells showing an increased capacity for production of IL‐4 [8] compared to CXCR3^hi^ T_FH_ cells that could represent a Th1‐biased T_FH_ cell subset [24, 25]. Overall, CD3^hi^PD1^hi^CD57^hi^CXCR3^lo^ T_FH_ cells were localised mainly in the inner zone/centre of the follicular areas, while CD3^hi^PD1^hi^CD57^lo^CXCR3^hi^ were found in the boundaries of the GC area (Figures 3C and S4A). When the number of identified cells permitted, further analysis of these two T_FH_ subsets, taking into consideration the expression of TIGIT and Bcl6, was applied. A differential localisation between the CD3^hi^PD1^hi^CD57^lo^CXCR3^hi^TIGIT^hi^Bcl6^lo^ and CD3^hi^PD1^hi^CD57^lo^CXCR3^hi^TIGIT^lo^Bcl6^hi^ T_FH_ subsets was observed, with TIGIT^lo^Bcl6^hi^ cells being found closer to the inner zone of the follicles (Figures 3C and S4A). Given the differential localisation of these T_FH_ cell subsets, we sought to analyse their distribution profile across the follicular areas and the corresponding B cell/T_FH_ cell neighbouring profiles and asked whether these profiles differ between tonsils and reactive LNs. To this end, we calculated the ‘G function’, a surrogate for the scatteredness/dispersion of the distribution of a given cell subset, the Euclidean mean minimum distance between T_FH_ and GC B cell subsets and the cross‐G function of these distances (Figure S4B). Selected follicles with at least 20 cells of the relevant cell subsets were analysed from two tonsils and two reactive LNs. DZ (CD20^hi/dim^CD3^lo^Ki67^hi^Bcl6^hi^) B cells were characterised by a significantly higher dispersion in tonsils compared to LNs, while a similar profile between the two organs was observed for LZ (CD20^hi/dim^CD3^lo^Ki67^lo^Bcl6^hi^) cells (Figure S4C). A trend, although not significant, for higher dispersion of CD3^hi^PD1^hi^CD57^lo^CXCR3^hi^ T_FH_ cells in tonsils compared to LNs was observed (Figure S4C). In line with their localisation profile (Figure 3C), a significantly shorter minimum distance between DZ B cells and CD3^hi^PD1^hi^CD57^hi^CXCR3^lo^ compared to CD3^hi^PD1^hi^CD57^lo^CXCR3^hi^ T_FH_ cells was found in tonsils but not in LNs (Figure 3D, upper panel). Furthermore, a significantly longer distance between CD3^hi^PD1^hi^CD57^hi^CXCR3^lo^ T_FH_ and DZ B cells was found in LNs compared to tonsils (Figure 3D, upper panel). Estimation of the randomness of the calculated distance profiles (cross‐G function for the calculated minimum distance among two cell types) showed a clear trend for a higher degree of randomness for the distance profile between DZ B cells and CD3^hi^PD1^hi^CD57^hi^CXCR3^lo^ compared to CD3^hi^PD1^hi^CD57^lo^CXCR3^hi^ T_FH_ cells in LNs (Figure 3D, lower panel). A clear trend for higher randomness (*p* = 0.0593) of the distance between DZ B cells and CD3^hi^PD1^hi^CD57^hi^CXCR3^lo^ T_FH_ cells was also found in LNs compared to tonsils (Figure 3D, lower panel). Altogether, our data point to a distinct spatial organisation of T_FH_ cell subsets with certain parameters, like the distance to specific follicular B cell subsets, of this organisation differing between tonsils and LNs.

### 3.4 Differential Prevalence of Specific T_FH_ Cell Subsets Between Tonsils and Reactive LNs

**FIGURE 4:**
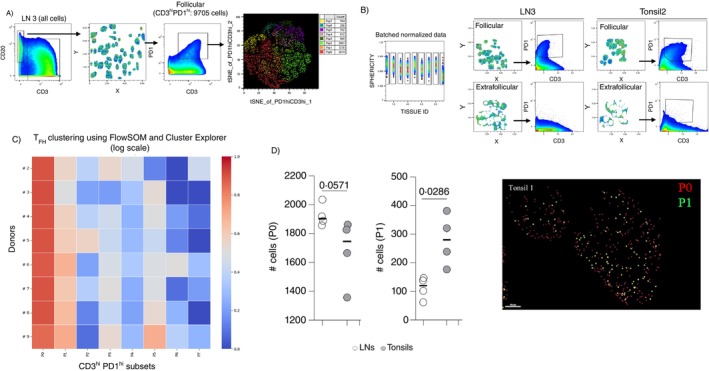
Identification of T_FH_ cell subsets differentially represented between tonsillar and LN tissues. (A) The histocytometry gating scheme for the identification of CD3^hi^PD1^hi^ T_FH_ cells in the total follicular area of an LN and their t‐SNE analysis using the biomarkers of our panel is shown. (B) Imaging data from LNs (*n* = 5) and tonsils (*n* = 4) were used in a batch analysis of T_FH_ cells. The gating scheme for the identification of CD3^hi^PD1^hi^ T_FH_ cells (the corresponding staining of the matched extrafollicular area is shown for comparison) for one LN and one tonsil is shown. (C) Heatmap showing the prevalence (judged by absolute cell counts) of FlowSOM‐identified T_FH_ cell subsets (P0–P7) in each tissue investigated (LNs, #2–5 and tonsils, #6–9). (D) Dot plot graph showing the cell counts of the P0 and P1 subsets in tonsils (*n* = 4, open circles) and LNs (*n* = 4, grey circles) (left). The relative positioning of the two T_FH_ cell subsets across a follicle is also shown (right). Each circle represents a different tissue sample. Statistical analysis was performed using the Mann–Whitney test, and *p* values are listed.

Given the in situ phenotypic heterogeneity of T_FH_ cells, we further analysed our imaging data from tonsils (*n* = 4) and LNs (*n* = 5) by using computational dimensionality reduction and clustering tools. Areas exhibiting background staining were filtered out. We should emphasise that the majority, if not all, of the non‐specific signal (likely due to non‐specific binding of used antibodies) was found in extrafollicular areas (e.g., tissue edges). The total follicular areas and CD3^hi^PD1^hi^ T_FH_ cells were identified for all tissues under investigation (Figures 4A and S5A). CD3^hi^PD1^hi^ T_FH_ cells were further analysed by applying unsupervised clustering FlowJo10 modules (t‐SNE, FlowSom and Cluster Explorer) to individual tissues. The analysis revealed the presence of several T_FH_ cell subsets (Figures 4A and S5B). To perform a ‘batch’ analysis of all tissues used, identified segmented cells from each tissue were assigned to a unique identifier (tissue ID), and a normalisation step of the intensities of all biomarkers used was applied (Figure 4B). Concatenating the same number (*n* = 2200) of T_FH_ cells from all samples used retains the capability for the detection of follicular regions and T_FH_ cell subsets in each tissue‐ID (Figure 4B) while unifying the downstream clustering analysis (Figures 4C and S6A–C). One LN was excluded from the batch analysis due to its low number of T_FH_ cells. Analysis of the identified T_FH_ cell subpopulations (Figure 4D, left panel and Figure S5B) showed significantly different prevalence of the subsets P0 (characterised by basal expression for several of the analysed molecules) and P1 (characterised by relatively increased expression of CD57 and low expression of TCF1 and TIGIT) between tonsils and LNs (Figure 4D, left panel). These two subsets were characterised by different localisation across the follicular areas (Figure 4D, right panel and Figure S6D). Therefore, our approach can dissect the T_FH_ cell phenotypic heterogeneity by identifying (i) T_FH_ cell subsets with altered prevalence among tissues of different origin as well as (ii) their localisation across the follicular areas.

### 3.5 In Situ Functional Heterogeneity of Human Tonsillar T_FH_ Cells

**FIGURE 5:**
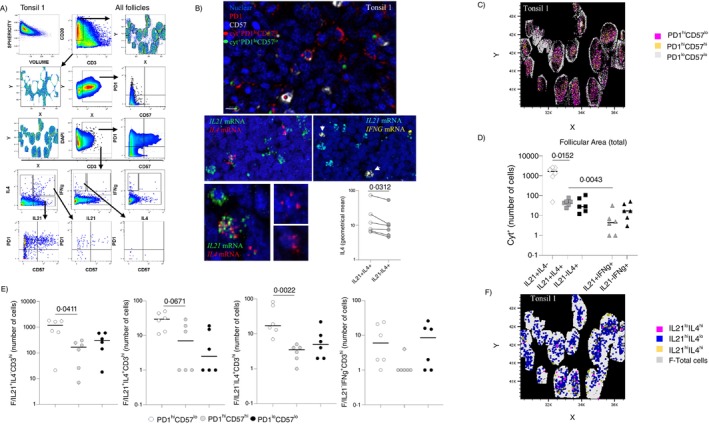
Investigation of the in situ functional heterogeneity of T_FH_ cells. (A) The histocytometry gating scheme for the identification of cyt^+^ T_FH_ cells and their phenotype with respect to PD1 and CD57 expression is shown. (B) Cyt^+^ cell spheres identified by histocytometry were backgated into the original mIF image. Their alignment to their original counterparts is shown (upper panel). Representative mIF images showing the expression of *IL21*, *IL4* and *IFNG* transcripts in follicular areas from tonsils. The geometrical mean intensity of *IL4* in the *IL21* ^+^ *IL4* ^+^ and *IL21* ^−^ *IL4* ^+^ T_FH_ cells is shown too (lower panel). (C) Digital representation of the location of T_FH_ cell subsets identified based on the expression of PD1 and CD57. (D) Dot plots demonstrating the number of CD3^hi^PD1^hi^ cell subsets, that produce different cytokine combinations, are shown. Each symbol represents a different tonsil (*n* = 6), and different symbols represent a different combination of expressed cytokine mRNA species. (E) Dot plots showing the numbers of T_FH_ cell subsets, identified based on the expression of PD1 and CD57, expressing the cytokine combinations shown. (F) Digital representation of the location of T_FH_ cells expressing the cytokine combinations shown. Statistical analysis was performed using the Wilcoxon (C, lower panel) or the Mann–Whitney (E and F) test, and *p* values are listed.

Development of T_FH_ cells is a multistep process associated with differential function/cytokine production (IL‐21 and IL‐4) at different phases [9]. We also developed a multiplex RNAscope/protein imaging assay allowing for the simultaneous detection and analysis of IL21, IL4, and IFNγ mRNA species to further equip our in situ armamentarium (Figure S7A). Preliminary experiments using tonsil tissues and RNAse treatment verified the specificity of probe staining (Figure S7B). Filtering out false‐positive events not associated with intact cells, by using their geometrical features, was applied when needed (Figure S7C). As expected, Histocytometry analysis showed that the majority of follicular *IL21* and/or *IL4* positive CD3^hi^ cells were expressing a PD1^hi^ phenotype (Figures 5A,B and S7D). Our gating revealed a considerable presence of CD3^hi^PD1^lo^CD57^lo^ T cells (Figure 5A) that were mainly located at the boundaries of the follicles, suggesting a less differentiated, pre‐T_FH_ stage (Figure 5C). Most of the *IL21* ^+^ cells express multiple transcripts (individual dots) of IL21, while this was less evident for the *IL4* or *IFNG* expressing cells (Figure 5B). Interestingly, the expression of *IL4* mRNA per cell (judged by the geometrical mean of fluorescence intensity) was higher in the *IL21* ^+^ *IL4* ^+^ compared to *IL21* ^−^ *IL4* ^+^ cells (Figure 5B, lower panel). A heterogeneity of T_FH_ cells was also observed with respect to their cytokine profile. Most of the cytokine‐expressing (cyt^+^) cells were single expressors of *IL21*, followed by *IL4* and *IL21/IL4* expressing cells (Figure 5D). The prevalence of follicular cells expressing *IFNG* was significantly lower compared *to IL21* or *IL4* expressing cells (Figure 5D). Interestingly, cells expressing both *IL21* and *IFNG* mRNA were found in three of the tissues tested (Figure 5D), while that was not the case for *IL4* and *IFNG* expression. Regarding the expression of PD1 and CD57 by cytokine‐expressing cells, we found that the PD1^hi^CD57^lo^ T_FH_ was the major IL21 and/or IL4‐expressing subset (Figure 5E). Follicular CD3^hi^ *IFNG* ^+^ T cells were accompanied either by a CD3^hi^PD1^hi^CD57^lo^ or CD3^hi^PD1^lo^CD57^lo^ phenotype (Figure 5E). In general, *IL21* ^+^ *IL4* ^+^ or *IL21* ^−^ *IL4* ^+^ cells were localised towards the inner circle of the follicles and exerted a more clustered phenotype compared to *IL21* ^+^ *IL4* ^−^ cells (Figures 5F and S7E). Conclusively, we revealed that the T_FH_ cell compartment is highly heterogeneous both at the phenotypic and functional levels.

### 3.6 Comparable Phenotypic Profile Between Follicular and Extrafollicular CD8 T Cells in Reactive LNs

**FIGURE 6:**
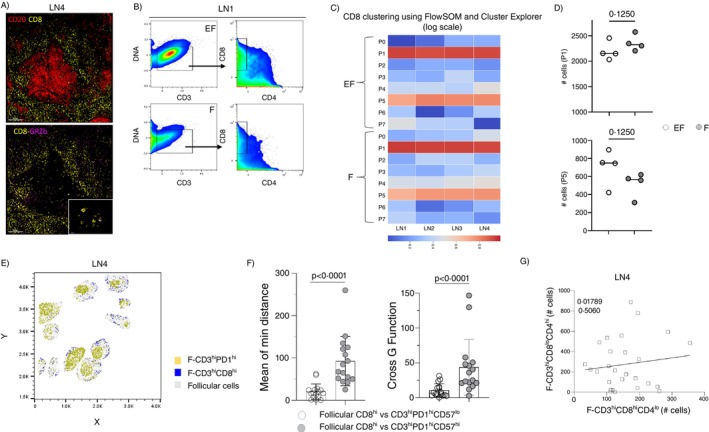
Similar phenotypic composition of follicular and extrafollicular CD8 T cells in reactive lymph nodes. (A) Representative mIF images showing the expression of CD20 (red), CD8 (yellow) and GrzB (magenta) in an LN (scale bar: 500 μm). Inserts show the CD8, GrzB staining pattern in a zoomed area. (B) The histocytometry gating scheme for the identification of CD3^hi^CD4^lo^CD8^hi^ T cells in the EF and total F of an LN. (C) Heatmap showing the prevalence (judged by absolute cell counts) of FlowSOM identified CD8 T cell subsets (P0–P7) in EF and F area for each LN tissue investigated (D) Dot plot graph showing the cell counts of the P1 and P5 subsets in EF (*n* = 4, open circles) and F (*n* = 4, grey circles) areas (right). Each circle represents a different LN donor (E) 2D histocytometry plot projecting the localisation of CD3^hi^CD4^lo^CD8^hi^ and CD3^hi^PD1^hi^ T_FH_ cells across follicular areas. Representative plots from one LN are shown. (F) Dot plot graphs showing accumulated data for the analysis of the minimum distance (left panel) between follicular CD3^hi^CD4^lo^CD8^hi^ cells and CD3^hi^PD1^hi^CD57^hi^ (open circles) or CD3^hi^PD1^hi^CD57^lo^ (grey circles) T_FH_ cell subsets and corresponding calculated cross‐G function (right panel). Each dot represents a follicle. Statistical analysis was performed using the Wilcoxon test, and *p* values are listed. (G) Follicular areas from four LNs were analysed, with each dot representing a follicle. The regression analysis between concatenated follicular CD3^hi^CD4^hi^CD8^lo^PD1^hi^ T_FH_ and follicular CD3^hi^CD4^lo^CD8^hi^ T cell counts, for all reactive lymph node follicles of one representative LN, is shown. The *R* and *p* values are listed.

Our imaging panel includes the CD8 and GrzB biomarkers (Figure 6A), allowing for the parallel analysis of T_FH_ and CD3^hi^ CD8^hi^T cells using the same set of biomarkers. A similar pipeline to the T_FH_ cell analysis was used for the analysis of CD3^hi^CD4^lo^CD8^hi^ T cells in follicular and extrafollicular areas of four LNs (Figures 6B and S8A). Analysis of the same number (*n* = 3000) of concatenated follicular and extrafollicular CD3^hi^CD4^lo^CD8^hi^ T cells, after normalisation of the biomarkers' intensity, showed a similar t‐SNE profile between the ‘F’ and ‘EF’ areas, for all LN tissues tested (Figure S8B). Then, the prevalence of identified subsets (using FlowSOM and Cluster Explorer) (Figures 6C and S8C,D) was compared between ‘EF’ and ‘F’ for four LNs. A similar profile was found between the two areas (Figures 6C,D and S8E). A regulation between T_FH_ and fCD8 T cells, mediated, for example, by IL21 [26] or the killing function of CD8 T cells [27], could impact the prevalence of both immune cell subsets, especially in the context of a disease [28]. A localisation of CD3^hi^CD4^lo^CD8^hi^ T cells towards the outer follicular area compared to T_FH_ cells was observed (Figure 6E). The calculation of minimum distances between CD8 T cells and two functionally distinct [27] T_FH_ cell subsets revealed a significantly longer distance between CD8 and CD57^hi^ compared to CD57^lo^ T_FH_ cells (Figure 6F), in line with their localisation profile (Figures 6E and S3C). Analysis of CD3^hi^CD8^lo^CD4^hi^PD1^hi^ and CD3^hi^CD4^lo^CD8^hi^ T cells in each follicular area per donor showed no correlation in cell counts between these two immune cell types for all donors (*n* = 4) tested (Figure 6G). Together, our data suggest that the transition of CD3^hi^CD4^lo^CD8^hi^ T cells from the extrafollicular to follicular compartment does not appear to be accompanied by major phenotypic changes, based on the biomarkers analysed.

### 3.7 In Situ Analysis of Immune Cell Types and Mediators That Can Potentially Modulate GC‐Reactivity

**FIGURE 7:**
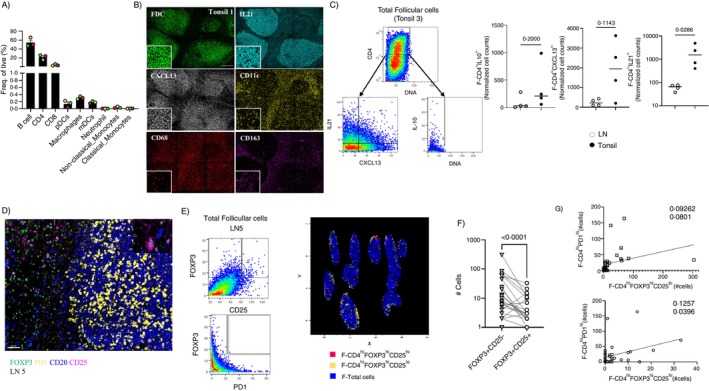
No significant differences between tonsillar and LN tissues with respect to innate immune cell subset prevalence. (A) Frequency of immune subsets from live gated cells. Colour code represents an individual (*n* = 3). (B) Representative mIF images showing the expression of FDC (green), IL21 (cyan), CXCL13 (grey), CD11c (yellow), CD68 (red) and CD163 (magenta) in one tonsil (scale bar: 300 μm). Inserts show the expression of individual biomarkers in a zoomed area. (C) The histocytometry gating scheme for the identification of F, EF areas and relevant cell subsets in a tonsil is shown. Dot plots showing the normalised cell counts of follicular CD4^hi^IL10^hi^, CD4^hi^CXCL13^hi^ and CD4^hi^IL21^hi^ T cells in LNs (*n* = 4, open circles) and tonsillar (*n* = 4, closed circles) follicular areas. Statistical analysis was performed using the Mann–Whitney *U*‐test. (D) Representative mIF images showing the expression of FOXP3 (green), CD25 (magenta), PD1 (yellow) and CD20 (blue) in one LNs. (E) 2D Histocytometry plots showing the gating scheme for the identification of follicular CD4^hi^CD25^lo^FOXP3^hi^ and CD4^hi^CD25^hi^FOXP3^hi^ cells as well as the co‐expression of FOXP3 and PD1 (left panel). The relative positioning of FOXP3^hi^CD25^lo^CD4^hi^ of CD4^hi^CD25^hi^FOXP3^hi^ T cells across the follicular areas is shown in the right panel. (F) Dot plot showing the absolute counts of CD4^hi^CD25^lo^FOXP3^hi^ and CD4^hi^CD25^hi^FOXP3^hi^ T cells in individual follicles from three LNs analysed. Data were analysed using the Wilcoxon test. (G) The regression analyses between T_FH_ and CD4^hi^CD25^lo^FOXP3^hi^ or CD4^hi^CD25^hi^FOXP3^hi^ T cells in individual follicles from three LNs analysed are shown. Each dot represents a follicle. The R and *p* values are also listed.

Follicular innate immunity cells like CD68^hi^ tingible body macrophages [29], dendritic cells CD11c^hi^(DCs) or CD123^hi^ (pDCs) [30] and endothelial cells (CD31^hi^) as well as cytokines like IL‐21 (produced by T_FH_ cells), CXCL‐13 (mainly produced by Follicular Dendritic Cells‐FDCs and T_FH_ cells) and IL‐10 (produced by several cells including macrophages, CD4 T cells and a subset of B cells‐ [31]) contribute to the orchestration of follicular and GC immune responses. Tonsillar‐derived cells were initially analysed by flow cytometry (Figures 7A and S9A). As expected, B cells were the dominant cell type, followed by CD4 and CD8 T cells (Figure 7A). Although at significantly lower frequency, dendritic cells (mDCs and pDCs) and macrophages were the most frequent cell types among the analysed innate immune cells (Figure 7A). Next, we developed an mIF assay that allows the identification and quantification of relevant cell types as well as IL‐21, CXCL‐13, and IL‐10 positive cells (Figures 7B and S9B). The histocytometry gating strategy for the identification of corresponding cell types is shown in Figures 7C and S9C. In tonsillar follicles, CD11c^hi^ DCs were the most abundant innate cell subset, followed by CD68^hi^ or CD163^hi^ macrophages, MPO^hi^ and CD123^hi^ cells (Figure S9D). No significant differences were found between tonsils and LN follicles with respect to the prevalence of innate immune cells, but a significant decrease of CD31^hi^ endothelial cell counts was observed in tonsillar follicles (Figure S9D). A trend, although not significant, for higher cell density of bulk follicular IL10^hi^ was found in LNs compared to tonsils (Figure S9E). However, the analysis of follicular and extrafollicular CD4^hi^ IL10^hi^T cells showed the opposite profile (Figures 7C and S9F), possibly reflecting the significantly higher cell density of CD4 T cells in tonsils compared to LNs (Figure S9F). Both CD4^hi^IL21^hi^ T and CD4^hi^CXCL13^hi^ T normalised cell counts were higher in tonsillar follicles (although CD4^hi^CXCL13^hi^ results did not reach statistical significance) compared to LNs (Figure 7C). Conclusively, even if we did not observe any significant alterations of innate immune cell subsets, our data suggest that relevant follicular/germinal centre cytokines/chemokines may contribute, at least in part, to the increased GC‐reactivity in tonsil compared to LN follicles.

The in situ detection of another potential immunomodulatory cell subset, the follicular CD4^hi^FOXP3^hi^ (T_FR_) cells, was then addressed by an mIF assay (Figure 7D). Our analysis was focused on LN samples due to the low number of CD4^hi^FOXP3^hi^ cells in tonsils and revealed that the vast majority of CD4^hi^FOXP3^hi^ cells exhibited low or no expression of PD1 (Figure 7E). Relative position analysis indicated that CD4^hi^CD25^hi^FOXP3^hi^ cells were located towards the inner areas of the follicles compared to CD4^hi^CD25^lo^FOXP3^hi^ T cells (Figure 7E). We found consistently lower counts of CD4^hi^CD25^hi^FOXP3^hi^ compared to CD4^hi^CD25^lo^FOXP3^hi^ cells in most of the LN follicles analysed (Figure 7F). There was no correlation between T_FH_ and CD4^hi^CD25^lo^FOXP3^hi^, and a weak but significant correlation with CD4^hi^CD25^hi^FOXP3^hi^ T cells was observed in the reactive LN follicles (Figure 7G). Our data suggest that reactive, control LN follicular areas are populated with a considerable number of potential immunomodulatory T_FR_ cells that may shape GC reactivity alone or in combination with other immune cell subsets.

## 4 Discussion

We have used tonsils as a prototype organ for the study of follicular immune dynamics and compared them to reactive, cancer‐free, HIV‐free LNs. The development of GC immune responses against pathogens requires the coordinated function of several cell types, including stromal cells like FDCs and CXCL12‐expressing Reticular Cells‐CRCs, that provide survival factors and chemo‐attractants for T_FH_ and GC B cells [32, 33], adaptive and innate immunity cell types [1, 34, 35]. The architecture of the follicular areas (mantle zone‐MZ‐, DZ and LZ) enables the compartmentalisation of the immunereaction between GC cells, a critical factor for their coordinated function. Therefore, the spatial organisation shapes the interaction between relevant cell subsets and the development of mature GCs [36]. Presumably, loss of this organisation is associated with the pathogenesis/progression of diseases like chronic HIV/SIV infection [5]. Imaging methodologies allowing for the in situ characterisation of the phenotype, function and spatial organisation of GC cell subsets will help to further understand the cellular origin of such diseases. Here, we have employed mIF assays, complemented with computational analysis, to investigate the follicular immune landscape in human lymphoid organs. Using T_FH_/GC B cell biomarkers, we have developed an mIF panel able to reveal the in situ phenotypic heterogeneity of T_FH_ cells in line with the heterogeneity revealed by the analysis of tissue‐derived single‐cell analysis at either protein (CyTOF) or gene expression (scRNA) level. We should mention that all three approaches (CyTOF, scRNA and mIF) provided a similar profile of relative expression for individual markers (e.g., Tcf1, CD57 and Ki67) further validating our experimental approach. Furthermore, our mIF analysis helps for (i) the phenotypic identification of T_FH_ cell populations, (ii) their localisation in different areas within a given follicle and (iii) their heterogeneity with respect to their prevalence among different follicles within the same tissue. Previous studies suggested that T_FH_ cells can translocate among follicles within the same LN to maximise the capacity of the immune system for the formation of appropriate T_FH_/B cell interactions [37]. Our approach could be informative from that perspective, particularly for diseases where the follicular immune cellular composition can be dramatically affected [10, 38].

It has been proposed that a subset of T_FH_ cells (Th1‐like) that express CXCR3 [25] could play a role in shaping GC reactivity, possibly through the production of IFNγ [24, 39]. On the other hand, antigen‐specific T_FH_ cells able to produce IL‐4 are critical for the development of neutralising antibodies in vivo [40]. We have recently described that PD1^hi^CD57^hi^ T_FH_ cells are biased towards IL‐4 production, at least in vitro [8]. A T_FH_ cell subset, and associated signalling, is impaired in Systemic Lupus Erythematosus follicular areas [41]. In line with previous data [8], PD1^hi^CD57^hi^CXCR3^lo^ T_FH_ cells were localised closer to the inner zone of the tonsillar and LN GCs compared to PD1^hi^CD57^lo^CXCR3^hi^, further supporting the compartmentalisation of T_FH_ cell subsets based on their capacity for cytokine production. Likewise, CD3^hi^PD1^hi^CD57^lo^CXCR3^hi^TIGIT^lo^Bcl6^hi^ (GC‐) T_FH_ cells were localised towards the ‘centre’ of the GC compared to their CD3^hi^PD1^hi^CD57^lo^CXCR3^hi^TIGIT^hi^Bcl6^lo^ (pre‐GC) counterparts. Whether this T_FH_ cell subset that retains Bcl6 high expression is more skewed towards a Th2‐like T_FH_ phenotype merits further investigation. Additionally, the peripheral localisation of TIGIT^hi^ T_FH_ comes in agreement with previous reports that associate sustained TIGIT expression with a pre‐GC T_FH_ molecular and functional phenotype [42]. Presumably, the spatial organisation of cell types is also an indicator of the possibility for their interaction. We found a more dispersed profile of DZ‐B cells (CD20^hi^Ki67^hi^Bcl6^hi^) in tonsils compared to LNs. Whether this profile is due to differences in DZ size or the local distribution of signals (e.g., CXCL12) attracting LZ‐B cells to DZ needs further investigation. Contrary to DZ, a similar distribution profile was found for the LZ‐B cells, PD1^hi^CD57^lo^CXCR3^hi^ and PD1^hi^CD57^hi^CXCR3^lo^ T_FH_ cells between tonsils and LNs. Our data revealed a closer proximity, and presumably higher probability for interaction, between DZ‐ B cells and CD3^hi^PD1^hi^CD57^hi^CXCR3^lo^ compared to CD3^hi^PD1^hi^CD57^lo^CXCR3^hi^ T_FH_ cells, especially in tonsils. With respect to the randomness of the distance profiling, the profile between DZ‐B and CD3^hi^ PD1^hi^CD57^hi^CXCR3^lo^ T_FH_ cells was less random in tonsils compared to LNs. Assuming that CXCR3 marks Th‐1 biased T_FH_ cells, our data suggest that their interaction with follicular B cells may occur at the boundaries of MZ/GC during early stages of B cell differentiation compared to CD3^hi^PD1^hi^CD57^hi^CXCR3^lo^ T_FH_ cells which are closer to DZ‐B cells and possibly are more skewed towards a Th‐2 type of T_FH_ cells.

Our batch analysis allows for the direct comparison between tissues with respect to the prevalence of specific T_FH_ cell subsets. Normalising the raw intensity for each biomarker used, adjusting the mIF image coordinates and assigning the same ID to all cells from a given tissue allows all tissues to be processed as a ‘single merged’ sample. This minimises inconsistencies caused by the application of clustering algorithms compared to applying them to individual tissues separately. The comparison between tonsils and LNs revealed the presence of two T_FH_ subsets with significantly different prevalence. Interestingly, the P1 subset is characterised by a CD57^hi^ phenotype. Furthermore, our pipeline allows for the simultaneous analysis of the localisation of T_FH_ subpopulations of interest. Therefore, the in situ characterisation of T_FH_ cell heterogeneity can be extended to the identification of specific T_FH_ cell subsets that may be affected by a disease as well as the impact of the disease on their spatial localisation.

Our previous studies suggested a link between T_FH_ cell phenotype, molecular profile, function, and spatial positioning [8]. Our multiplex RNAscope analysis showed that IL‐21 is the most abundant cytokine produced by T_FH_ cells, while their phenotypic heterogeneity is also associated with a functional heterogeneity with respect to the combination of the produced cytokines and the localisation of the corresponding T_FH_ cell subset. Extrafollicular or perifollicular (secreted, at least in part, by PD1^lo^CD57^lo^ cyt^+^ cells) IL‐21 and IL‐4 may support the initial activation, differentiation of CD4 T cells and B cells by upregulating critical transcriptional factors like Bcl6 [9] while within the GC, IL‐21 could further support T_FH_/B interaction [43] and IL‐4 promote the differentiation and maturation of B cells [44, 45]. We observed an overall tendency for clustering of *IL4* ^+^ compared to *IL4* ^−^ cells in the inner areas of the follicle, further supporting the role of IL4 in later stages of B cell maturation. Although in smaller numbers, T_FH_ cells expressing *IFNG* mRNA were detected, which may represent Th1‐like T_FH_ cells. Whether these cells are also Tbet^hi^ [46] needs to be investigated. Our data can fuel further development of mIF RNAscope assays, including biomarkers for surrounding cells, to understand the possible impact of the microenvironment on T_FH_ cell functionality.

Diseases such as chronic HIV/SIV are associated with significant accumulation of follicular CD8 T cells [10, 47]. These immune dynamics are poorly understood in health. Our approach allows for the direct comparison of T_FH_ and fCD8 T cells within the same follicular/GC areas. The comparison between follicular and extrafollicular areas identified CD8 T cell subsets exhibiting a similar profile between the two areas, at least for the set of markers used. We have previously reported different transcriptomic signatures between extrafollicular and follicular CD8 T cells in HIV [10]. Therefore, the extension/update of the mIF biomarkers could address the dynamics of specific CD8 T cell subsets between the two anatomical compartments and their possible role in disease pathogenesis. The localisation profiling and distance analysis suggest a differential interaction between CD8 and T_FH_ cells across the follicular subareas, with the CD57^lo^ T_FH_ cell subset more likely to be affected by the CD8 function, at least under physiological conditions. Our correlation analysis showed no association between bulk T_FH_ and fCD8 T cells in LNs, suggesting independent dynamics (differentiation, trafficking) for these two follicular cell subsets in health. The comparison between follicular and extrafollicular areas identified CD8 T cell subsets exhibiting a similar profile between the two areas. Our approach provides an immunological basis for investigating the effect of disease on follicular CD8, T_FH_ cell prevalence, localisation and potential interaction.

In addition to follicular CD8 T cells, innate immunity cells and potential immunomodulatory follicular CD4 T cells (T_FR_) represent additional immune regulators of the GC development and reactivity. Analysis of innate immune cells using a second mIF assay showed readily respectable numbers of cells, such as macrophages and dendritic cells, in both tonsils and LNs. However, our analysis showed comparable cell densities of innate cell subsets between the two organs. Notably, the significantly higher cell density of the follicular IL21^hi^ (and CXCL13^hi^) CD4 T cells observed in tonsils may reflect a higher magnitude of GC reactivity in tonsils compared to LNs. With respect to T_FR_ cells, we observed a considerable number of cells in LNs, while few events were found in tonsils (data not shown), which is in line with our previous data [48]. Our data showed a dissociation between PD1 and FOXP3 expression in LNs suggesting that the CD4^hi^CD25^lo^FOXP3^hi^ T cells may not represent T_FH_ cells that adopt a FOXP3^hi^ phenotype and may act as regulators of GC size [49]. In addition to significantly different prevalence, a differential localisation between CD25^hi^FOXP3^hi^ and CD25^lo^ FOXP3^hi^ T_FR_ was also revealed. Overall, our data indicate a differential localisation of T_FH_ cell subsets, which may also be associated with a differential localisation of T_FR_ cells. Whether this profile translates into differential impact of T_FR_s on specific T_FH_ subsets requires further investigation.

Altogether, we have established an experimental approach for the comprehensive in situ characterisation of human follicular immune cells in health. The main goal of the study was to present such an approach applied to a relatively small cohort of tonsils and reactive LNs. Its application to a larger number of relevant tissues is needed for the validation/confirmation of the described profile. It should be emphasised that the described profiles (phenotypic heterogeneity, prevalence of individual subsets) of T_FH_ and fCD8 T cells are based on the analysis of the specific biomarkers used. High‐throughput methodologies like scRNA analysis of tissue‐derived cells can reveal novel molecules differentially expressed between health and disease or among different diseases. This could guide the development of similar mIF assays leading to the generation of disease‐specific tissue ‘signatures’ with respect to T_FH_ or fCD8 T‐cell in situ dynamics.

### 4.1 Limitations of the Study

Here, we developed and applied a multiplex imaging pipeline combined with relevant computational tools to characterise the follicular microenvironment of secondary lymphoid organs. Despite the statistical significance observed for several comparisons, we should mention the limited number of lymphoid tissues analysed. Furthermore, our descriptive in situ analysis should be combined with ex vivo functional studies to validate (or not) the impact of molecules/cell subsets analysed. Our data, however, provide proof of concept of the efficacy and potential of our experimental approach. Applying this approach to pathological conditions (chronic infections, cancer, autoimmunity) may provide more robust results.

## Author Contributions

S.G. and M.O. performed imaging experiments, analysed, interpreted imaging data and edited the manuscript. C.F. performed and analysed CyTOF experiments. C.B. and S.B. performed distribution analysis. H.L. performed scRNA analysis. G.X.M., F.R.B. and S.P.R. performed and analysed flow cytometry experiments. R.G. oversaw the scRNA analysis. G.P. assisted with the immunological data interpretation and the manuscript preparation. C.P. conceived, designed and supervised the study, interpreted data and wrote the manuscript. All authors have read, edited and approved the final version for submission.

## Conflicts of Interest

R.G. has received consulting income from Takeda, Sanofi, and declares ownership in Ozette Technologies and Modulus Therapeutics. The other authors declare no conflicts of interest.

## Supporting information


**Figure S1.** CyTOF, transcriptomic and mIF approaches for analysis of T_FH_ heterogeneity.
**Figure S2.** T_FH_ cell heterogeneity identified by histocytometry analysis of imaging data.
**Figure S3.** T_FH_ and B cell subsets protein expression and localisation profile examples in tonsils and LNs.
**Figure S4.** Analysis of localisation and distribution profiles of T_FH_ and B cell subsets in follicular areas.
**Figure S5.** Identification of T_FH_ cells in tonsillar and LN tissues used for further analysis by FlowJo10 plugins.
**Figure S6.** Analysis of normalised, concatenated T_FH_ cell data using FlowJo10 plugins.
**Figure S7.** Development of RNAscope mIF assay.
**Figure S8.** Analysis of CD3^hi^CD4^lo^CD8^hi^ T cells using FlowJo10 plugins.
**Figure S9.** Analysing innate immune cells and associated cytokines/chemokines.

## Data Availability

Data reported in this paper will be shared by the lead contact upon request. This paper does not report original code. Any additional information required to reanalyse the data reported in this paper are available from the lead contact upon request. The authors agree to share all publication‐related data. Presented data are accessible through 10.5281/zenodo.15280733. For further information, please contact the corresponding author at konstantinos.petrovas@chuv.ch.
