# Supplementary material for: Delineation of the Human Germinal Centre Immune Landscape Using Multiplex Imaging Analysis
Source: Immunology. 2025 May 27;176(1):87–104. doi: 10.1111/imm.13955 (PMC12318895; doi:10.1111/imm.13955)
Supplement: Supplementary file 1 — Figure S1. CyTOF, transcriptomic and mIF approaches for analysis of TFH heterogeneity. Figure S2. TFH cell heterogeneity identified by histocytometry analysis of imaging data. Figure S3. TFH and B cell subsets protein expression and localisation profile examples in tonsils and LNs. Figure S4. Analysis of localisation and distribution profiles of TFH and B cell subsets in follicular areas. Figure S5. Identification of TFH cells in tonsillar and LN tissues used for further analysis by FlowJo10 plugins. Figure S6. Analysis of normalised, concatenated TFH cell data using FlowJo10 plugins. Figure S7. Development of RNAscope mIF assay. Figure S8. Analysis of CD3hiCD4loCD8hi T cells using FlowJo10 plugins. Figure S9. Analysing innate immune cells and associated cytokines/chemokines. [file IMM-176-87-s001.docx]

**Delineation of the human germinal center immune landscape using multiplex imaging analysis.**

Spiros Georgakis^1^, Michail Orfanakis^1^, Craig Fenwick^2^, Cloe Brenna^1^, Simon Burgermeister^1^, Helen Lindsay^3^, Giuliana Xavier de Medeiros ^4^, Fernanda Romano Bruno^4^, Susan Pereira Ribeiro^4,5,6^, Raphael Gottardo^3^, Giuseppe Pantaleo^2^, Constantinos Petrovas^1*^.

^1^Institute of Pathology, Department of Laboratory Medicine and Pathology, Lausanne University Hospital and Lausanne University, Lausanne, VD, Switzerland.

^2^Service of Immunology and Allergy, Department of Medicine, Lausanne University Hospital and Lausanne University, Lausanne, VD, Switzerland.

^3^Biomedical Data Science Center, Lausanne University Hospital and Lausanne University, Lausanne, VD, Switzerland.

^4^Pathology Advanced Translational Research Unit (PATRU), Department of Pathology and Laboratory Medicine, Emory University School of Medicine, GA, USA.

^5^Emory Vaccine Center, Atlanta, GA, USA.

^6^Winship Cancer Institute of Emory University, Atlanta, GA, USA.

**Corresponding author**

Constantinos Petrovas, PhD

**Supplemental Figures**

**
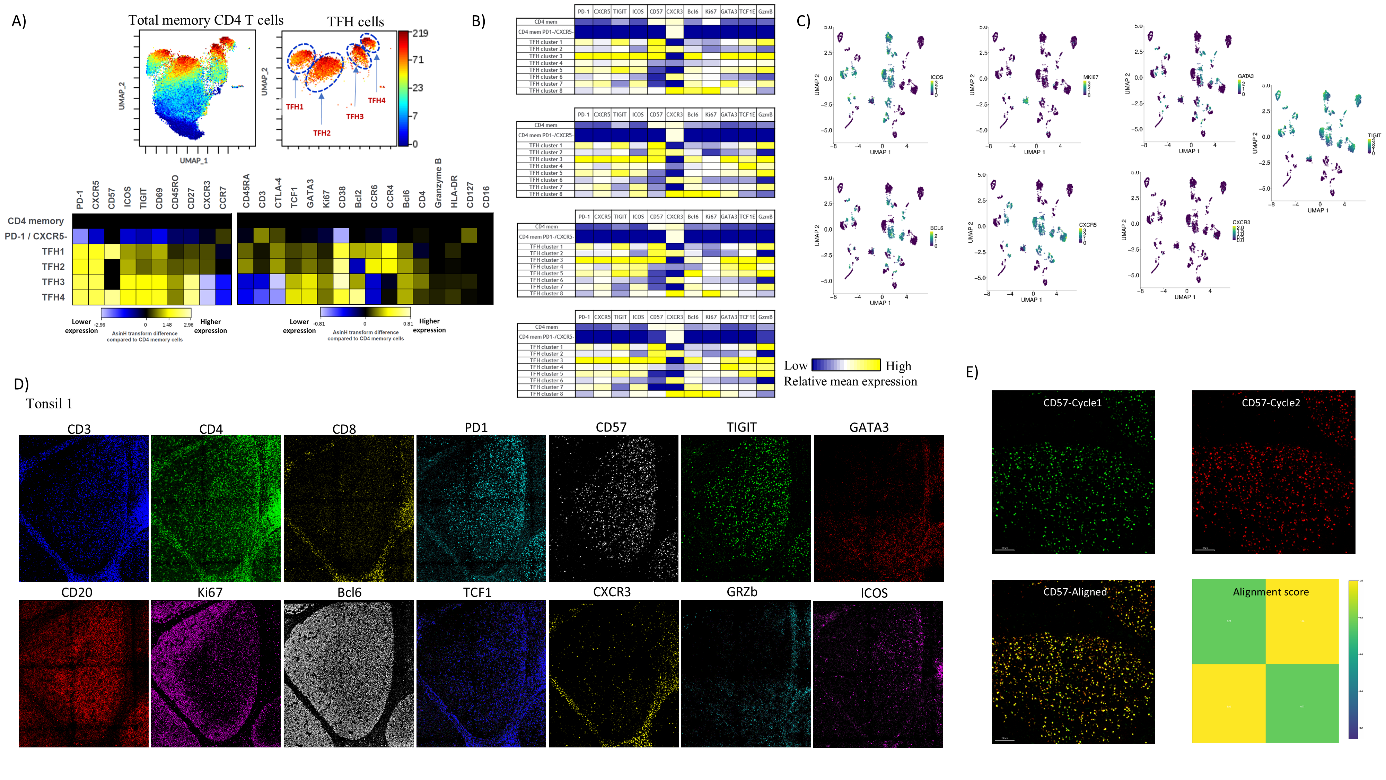
**

**Suppl. Figure 1. CyTOF, Transcriptomic and mIF approaches for analysis of T_FH_ heterogeneity. (A)** UMAP analysis showing the clustering of total memory CD4 T cells, for all four tonsils used (upper left panel) and the corresponding T_FH_ cell clusters (upper right panel). A heatmap showing the expression of all biomarkers used in total memory CD4, non-T_FH_ and T_FH_ cells from each tonsil (lower panel). A color indicator is provided **(B)** Heatmaps showing the relative mean expression of selected protein markers across different CD4 T and T_FH_ subsets from four individual tonsils. A color indicator is provided. **(C)** UMAPs highlighting the expression of selected genes in different tonsillar T_FH_ clusters derived from five tonsils **(D)** Representative mIF images showing the expression of individual biomarkers used for the T_FH_ imaging panel. Images from a tonsillar tissue are shown. **(E)** An example of the computational alignment of the images generated during the two cycles of the T_FH_ panel. CD57 was used as a ‘registration’ marker. The alignment score is also shown.


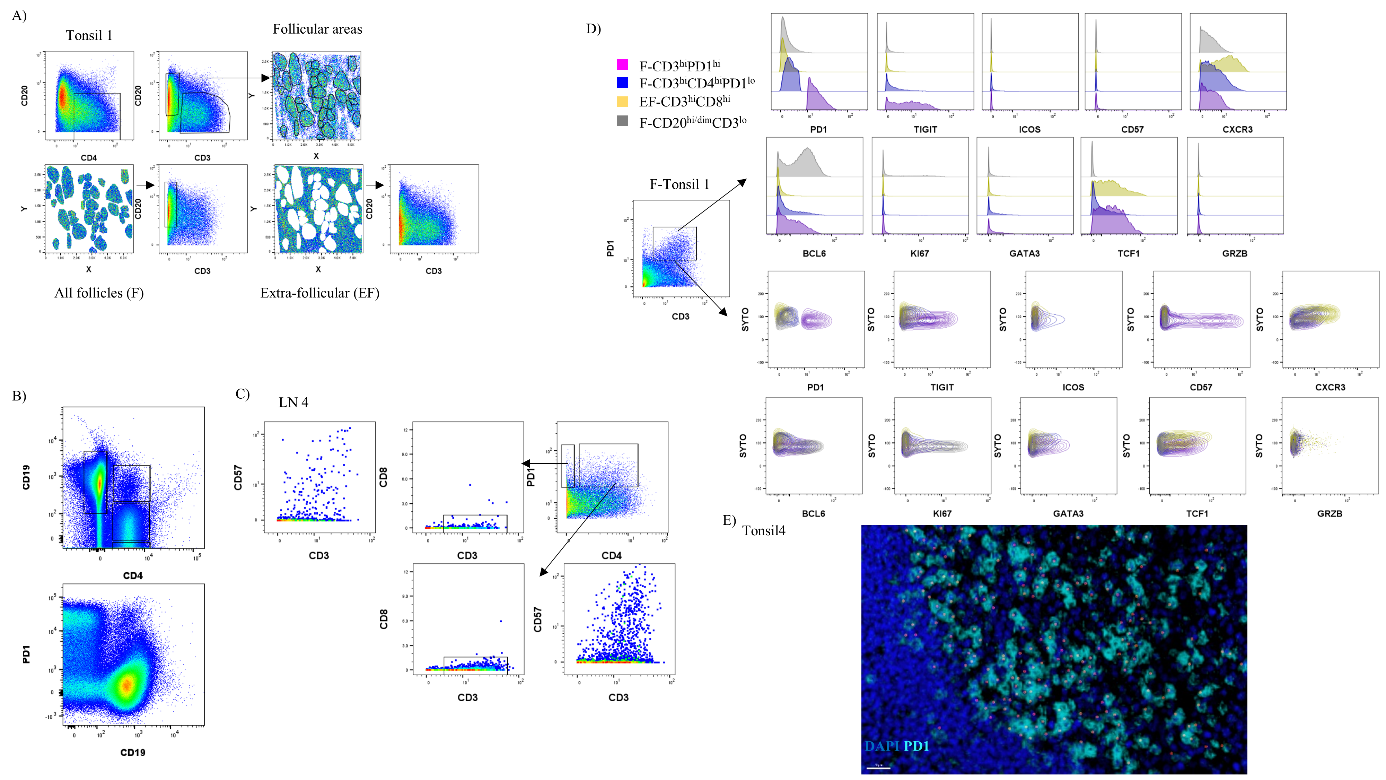


**Suppl. Figure 2. T_FH_ cell heterogeneity identified by Histocytometry analysis of imaging data. (A)** Histocytometry gating scheme for the identification of follicular, extrafollicular areas and CD3 and B cell subsets. **(B)** Flow cytometry analysis of tonsil derived single cell suspension. The gated population corresponds to double positive events for CD3 and CD19 (a B cell marker). **(C)** Histocytometry generated 2D plots showing the expression of PD1 vs CD4 in a LN as well as the downstream analysis of CD3, CD8 and CD57 expression. **(D)** The expression of individual biomarkers in CD3^hi^ PD1^hi^ T_FH_ cells from a tonsil as histograms (upper two rows) or 2D contour plots (lower two rows) is shown. **(E)** Digitally (Histocytometry) identified CD3^hi^PD1^hi^ T_FH_ cells (red spheres) were backgated into the fluorescent image of a tonsillar follicle and their alignment with their original counterparts is shown.

**
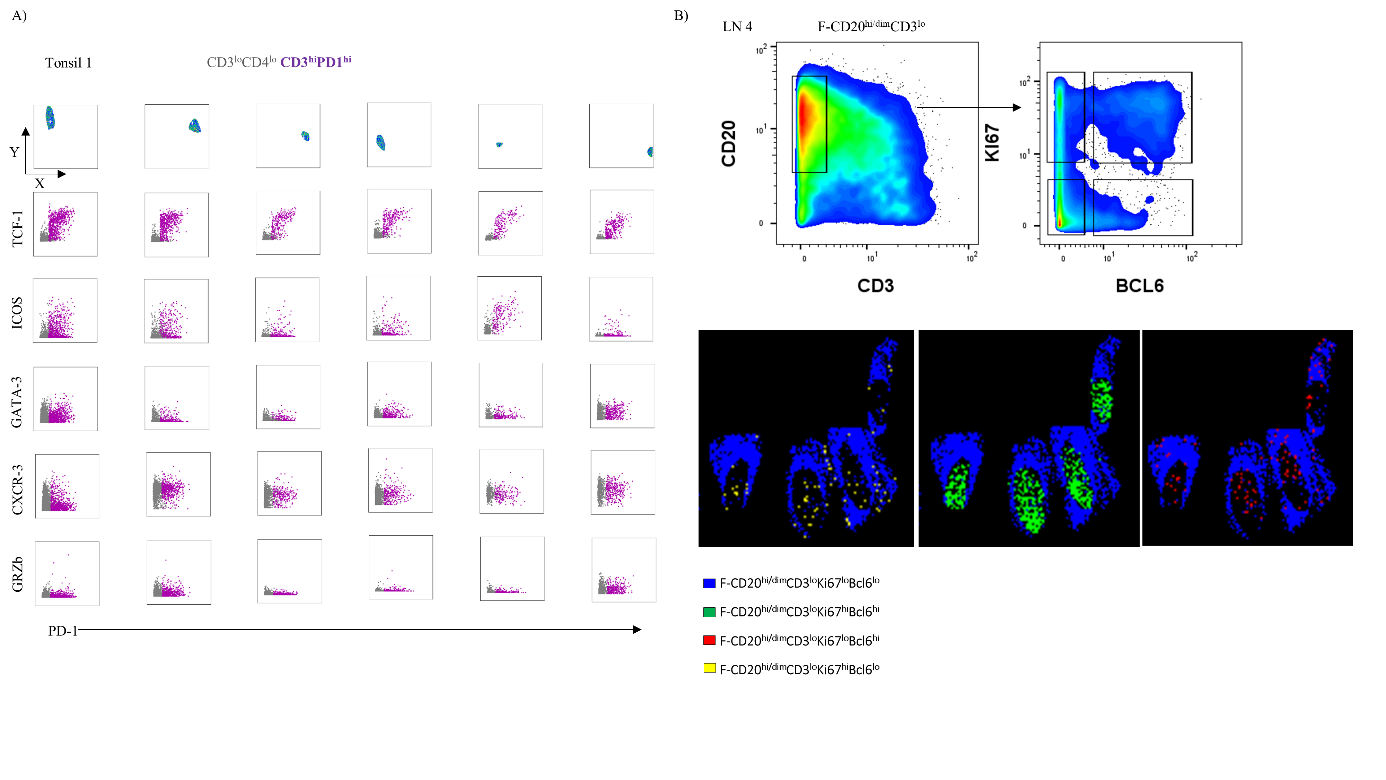
**

**Suppl. Figure 3.** **T_FH_ and B cell subsets protein expression and localization profile examples in tonsils and LNs. (A)** Six Histocytometry identified follicular areas from a tonsil (top row) and 2D contour plots showing the associated expression of biomarkers used vs PD1 in CD3^lo^CD4^lo^ (as reference cell subset) and CD3^hi^PD1^hi^ T_FH_ cells. **(B)** Histocytometry generated 2D plots showing the B cell subsets in a LN tissue (upper). The digital representation of their location is also shown (lower). 2D contour plots showing the expression of Ki67 vs Bcl6 in total follicular area and the corresponding localization of F-CD20^hi/dim^CD3^lo^Ki67^lo^BCl6^lo^ (blue-MZ), F-CD20^hi/dim^CD3^lo^Ki67^hi^BCl6^hi^ (green-DZ), F-CD20^hi/dim^CD3^lo^Ki67^lo^BCl6^hi^ (red-LZ) and CD20^hi/dim^CD3^lo^Ki67^hi^BCl6^lo^ (yellow).


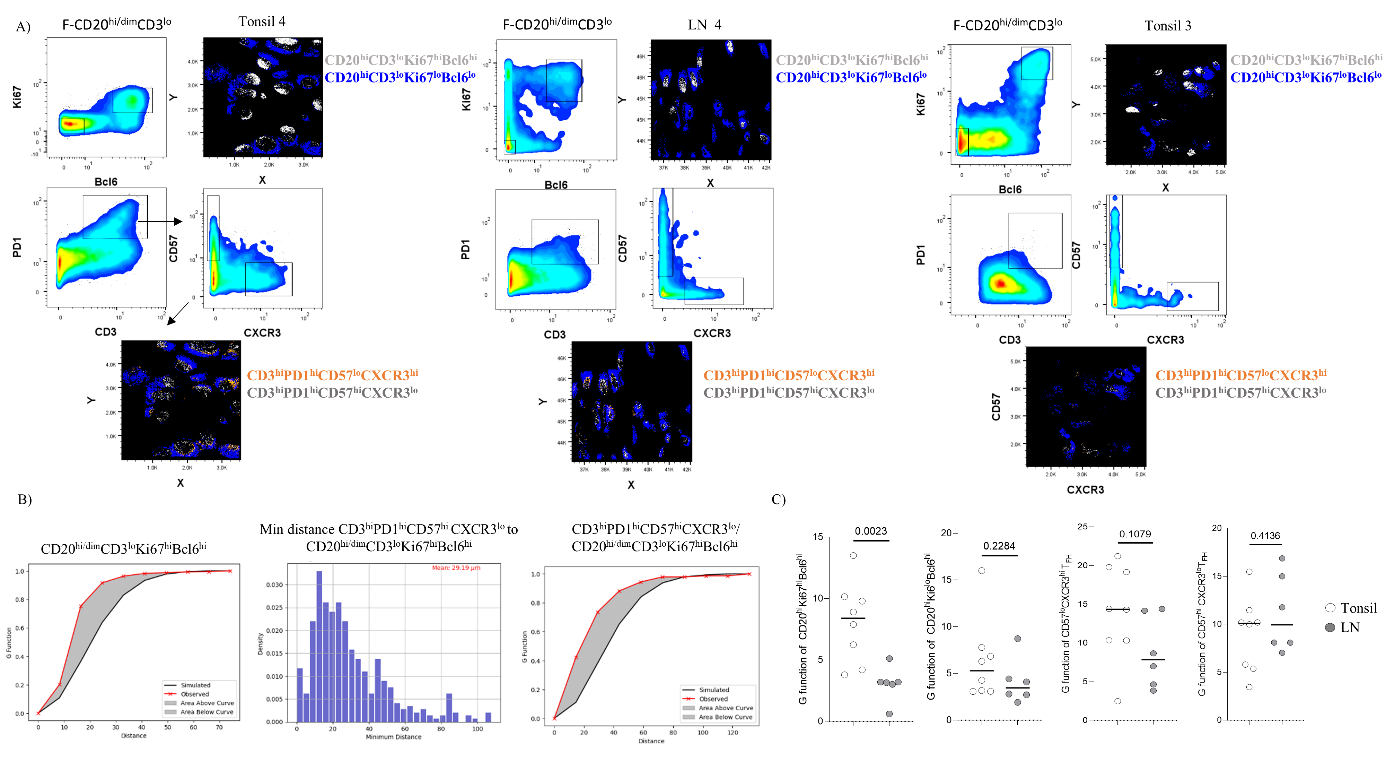


**Suppl. Figure 4. Analysis of localization and distribution profiles of T_FH_ and B cell subsets in follicular areas. (A)** 2D dot plots showing the expression of Ki67 vs Bcl6, CD3 vs PD1 and CD57 vs CXCR3 in total follicular area from one LN and two tonsils and the corresponding localization of CD20^hi/dim^CD3^lo^Ki67^lo^BCl6^lo^ (blue-MZ), CD20^hi/dim^CD3^lo^Ki67^hi^BCl6^hi^ (light grey-DZ), CD3^hi^PD1^hi^CD57^hi^CXCR3^lo^ (dark grey) and CD3^hi^PD1^hi^CD57^lo^CXCR3^hi^ (ceramide). **(B)** The spatial distribution of relevant cell subsets was assessed by the calculation of their corresponding G-Function. The proximity of the experimental G curve-red to the theoretical (Poisson) G curve-blue, as well as the gray area representing the area between the two curves measured are shown (left panel). The distribution bar graph for the minimum distance between CD3^hi^PD1^hi^CD57^hi^CXCR3^lo^ to CD20^hi/dim^CD3^lo^Ki67^hi^Bcl6^hi^ cells is shown in the middle. The experimental (red) and theoretical (blue) cross G function curves for the calculated minimum distances betweenCD3^hi^ PD1^hi^CD57^hi^CXCR3^lo^ and CD20^hi/dim^CD3^lo^Ki67^hi^Bcl6^hi^ cells are also shown (right panel). **(C)** Dot plots showing the G function (surrogate of distribution/ dispersion) for B and T_FH_ cell subsets in tonsils (n=7, open circles) and LNs (n=6, closed circles). Each dot represents a follicle. Statistical analysis was performed using Mann-Whitney test.


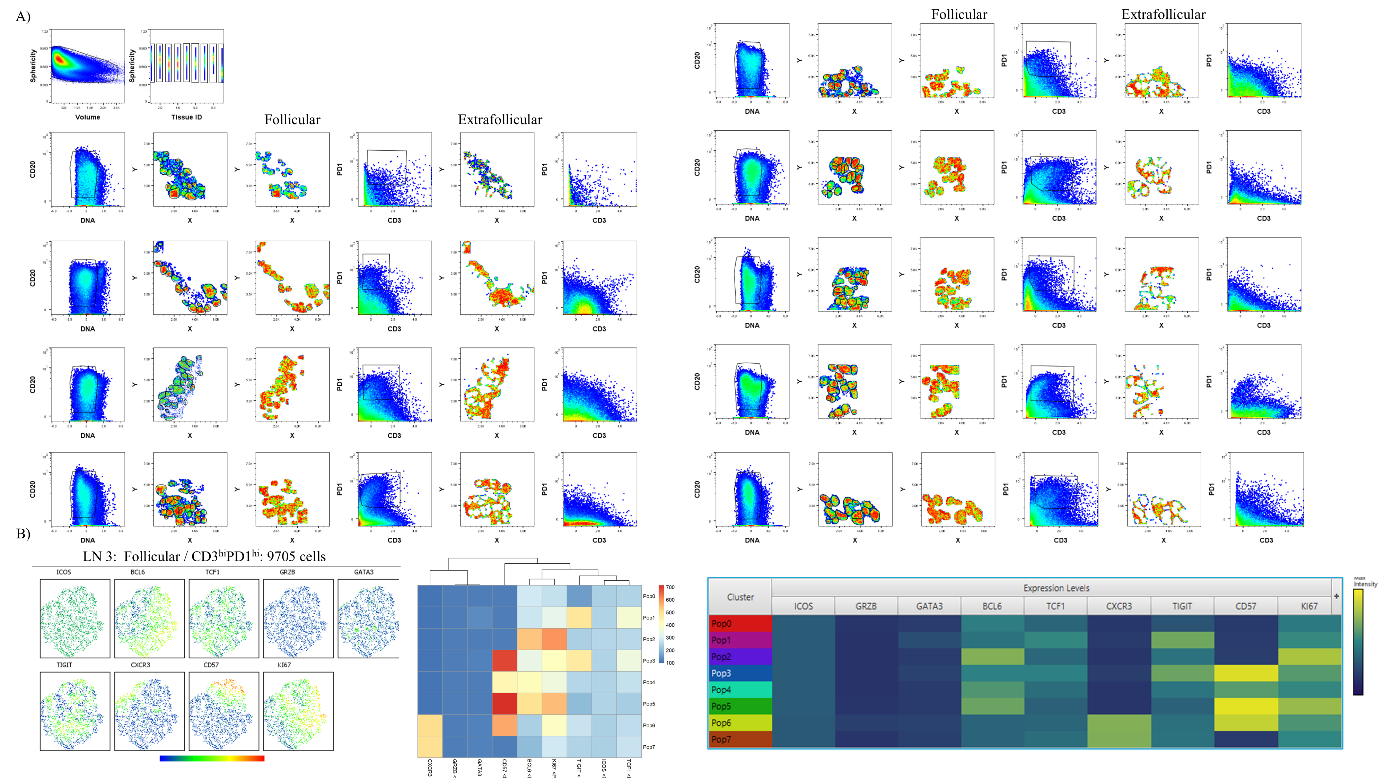


**Suppl. Figure 5. Identification of T_FH_ cells in tonsillar and LN tissues used for further analysis by FlowJo10 plugins (A)** Histocytometry gating scheme for the identification of total follicular, extrafollicular areas and CD3^hi^PD1^hi^ T_FH_ cells in each tonsil (left panel, n=4) and LN (right panel, n=5) used for downstream analysis using FlowJo10 plugins. Each row represents one tissue. **(B)** tSNE analysis of all CD3^hi^PD1^hi^ T_FH_ cells in the LN follicular area (left) and the associated FlowSOM (middle) and Cluster Explorer (right) generated heatmaps showing the identified eight subsets using the T_FH_ panel biomarkers. tSNE representation for the concatenated follicular CD3^hi^PD1^hi^ T_FH_ cells in all LNs (upper row) and tonsils (lower row) used.


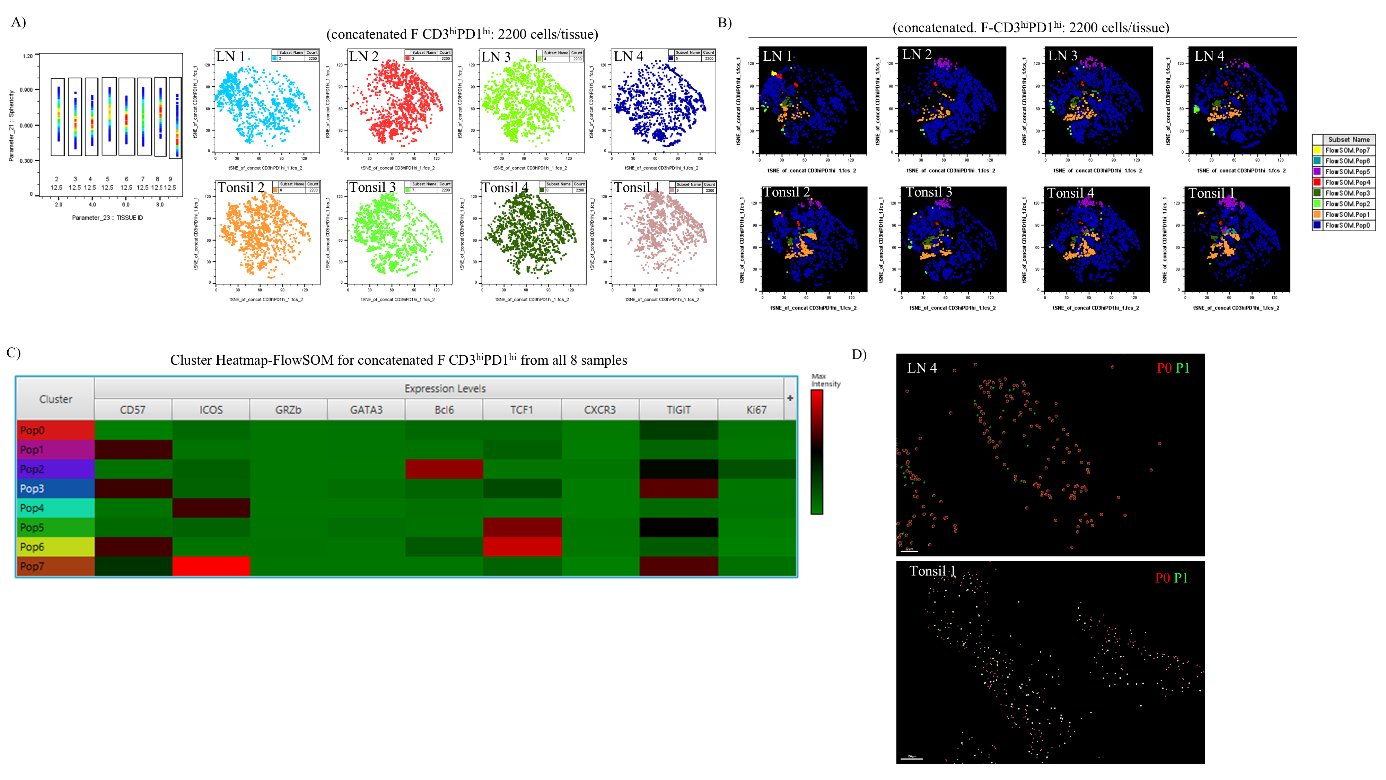


**Suppl. Figure 6. Analysis of normalized, concatenated T_FH_ cell data using FlowJo10 plugins. (A)** Imaging data form LNs (n=5) and tonsils (n=4) were used in a batch analysis of T_FH_ cells. tSNE representation for the concatenated follicular CD3^hi^PD1^hi^ T_FH_ cells in all LNs (upper row) and tonsils (lower row) used. **(B)** Equal number (n=2200) of T_FH_ cells from four LNs and four tonsils were concatenated and further analyzed using tSNE, FlowSOM and Cluster explorer FlowJ10 modules. The tSNE generated profiles for each tissue and the eight identified T_FH_ cell subsets are shown. The color identifier of individual cell subsets is also included. **(C)** Cluster Explorer generated heatmap showing the eight identified subsets for the follicular CD3^hi^PD1^hi^ T_FH_ cells from all used tissues. **(D)** Digital representation of the localization of subsets P0 and P1, identified by the clustering analysis, in representative follicles for one LN and one tonsil.


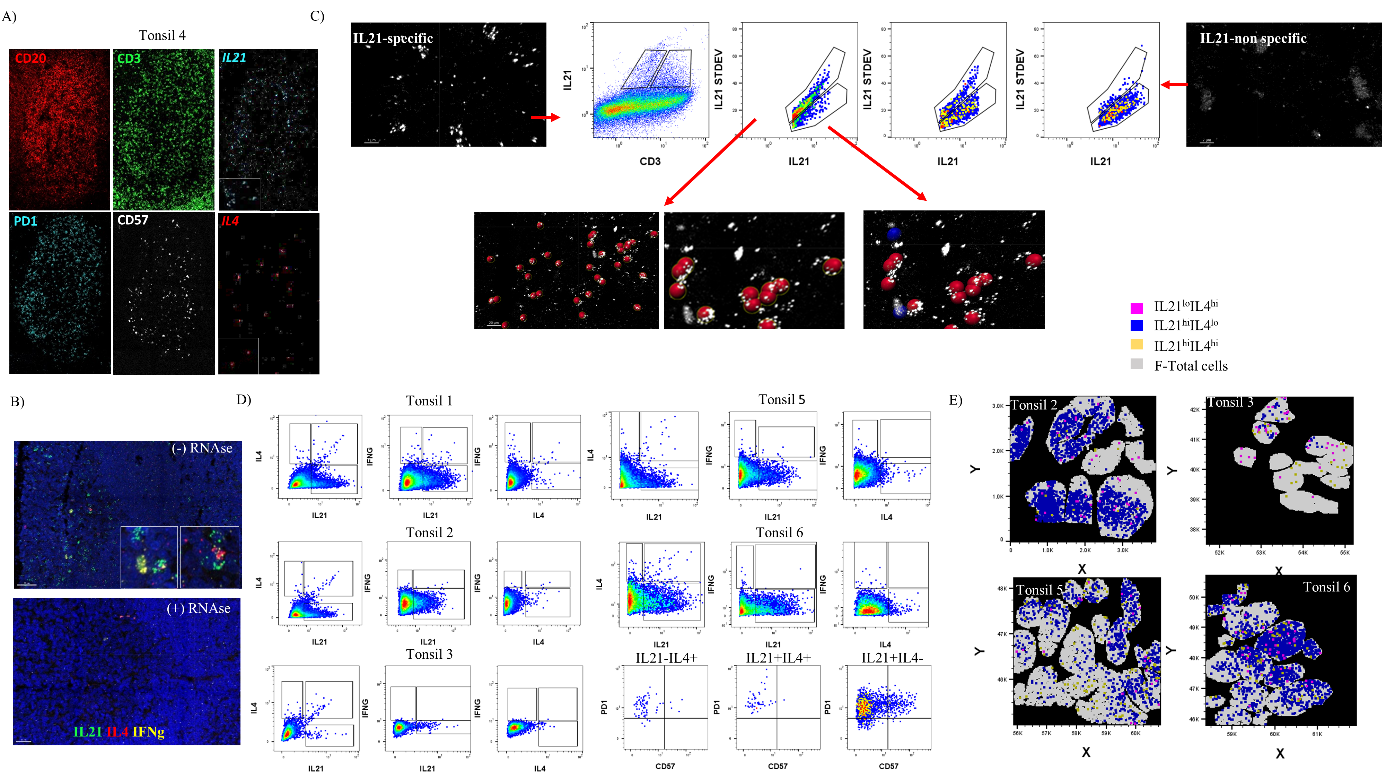


**Suppl. Figure 7. Development of RNAscope mIF assay. (A)** Immunofluorescence images showing the expression of CD20 (red), CD3 (green), PD1 (cyan), CD57 (white), *IL21* mRNA (cyan) and *IL4* mRNA (red) in a tonsillar follicle (scale bar.: 300μΜ). **(B)** RNAscope generated images showing the *IL21* mRNA signal in a tonsil without (upper) and after (lower) pretreatment of the tissue with RNase (scale bar: 10μm). Images were acquired using 63x. **(C)** The computational removal of background/non-specific positive events using Imaris modules is shown. Red spheres represent ‘real’ cyt^+^ cells while the blue spheres correspond to non-specific binding of the used probe. The 2D flow cytometry plots depict the expression of *IL21* with respect to the computationally calculated STDEV (standard deviation) of the *IL21* staining intensity per cell base. **(D)** Histocytometry generated 2D plots showing the expression of individual cytokines by T_FH_ cells in each tonsillar tissue used. The expression of PD1 and CD57 for the cyt^+^ T_FH_ cells in one tonsil is also shown (lower, right part). **(E)** Digital representation of the localization of cyt^+^ T_FH_ cells across the follicular areas (in gray) in four tonsillar tissues.


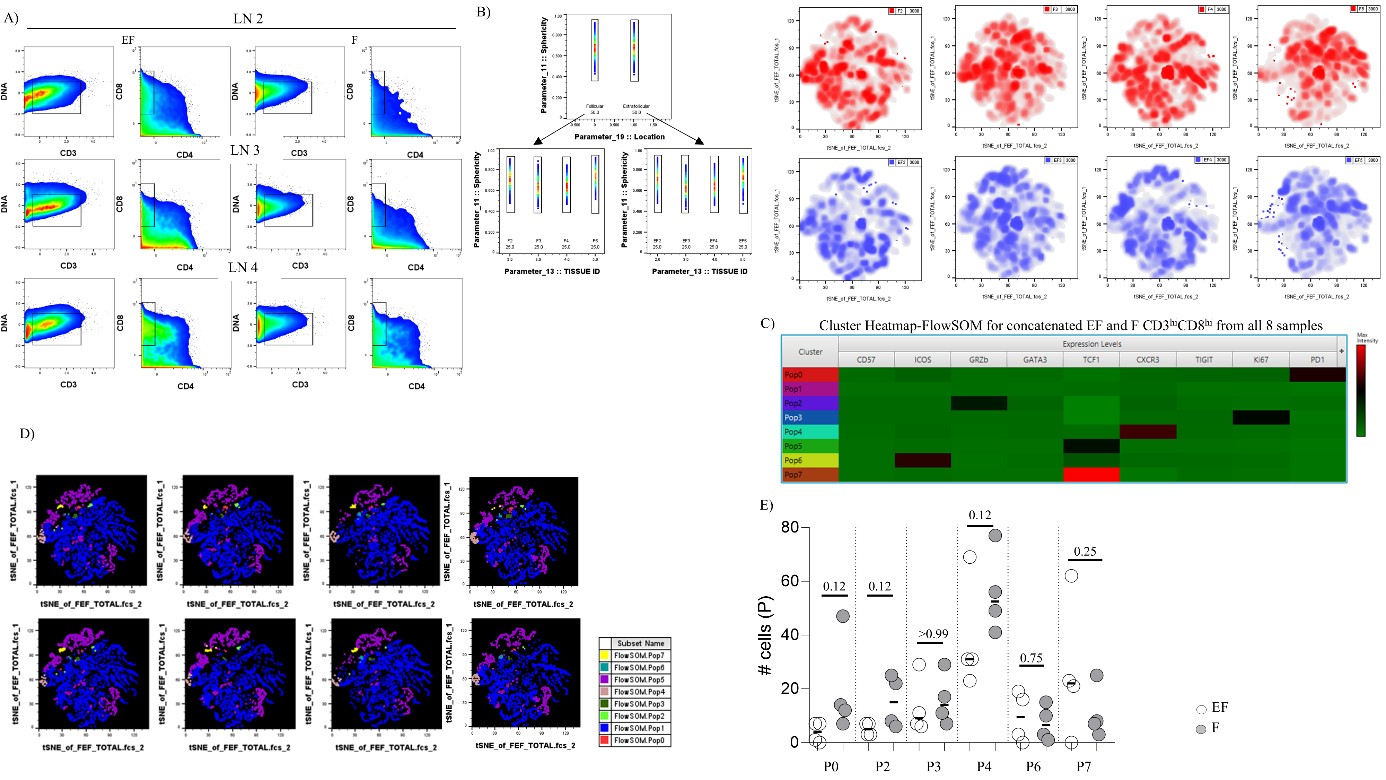


**Suppl. Figure 8. Analysis of CD3^hi^CD4^lo^CD8^hi^ T cells using FlowJo10 plugins. (A)** Histocytometry gating scheme for the identification of CD3^hi^CD4^lo^CD8^hi^ T cells in the extrafollicular (EF) and follicular (F) areas from three of the used LNs. **(B)** Imaging data from LNs (n=4) were used for batch analysis comparing EF- to F- CD8 T cell subsets. Normalized intensities of relevant biomarkers were used. Equal number (n=3000) of CD8 T cells were used for concatenation and downstream analysis of cell subsets using tSNE, FlowSOM and Cluster Explorer modules (FlowJo10). The identification of the EF- (n=4) and matched F- (n=4) populations are shown (left). tSNE representation for the EF- and F- CD3^hi^CD4^lo^CD8^hi^ T cells for the four used LNs (right). **(C)** Cluster Explorer generated heatmap showing the CD3^hi^CD4^lo^CD8^hi^T cell subsets identified and their relative protein expression (n=8). **(D)** tSNE representation of the eight subsets for the EF and F cells from the four LNs used. **(E)** Dot plot showing the cell counts for the CD3^hi^CD4^lo^CD8^hi^ T cell subsets P0, P2, P3, P4, P6, P7 in the EF and F areas for all eight tissues used. Each circle represents a different donor (EF=open circle, F=grey circle).


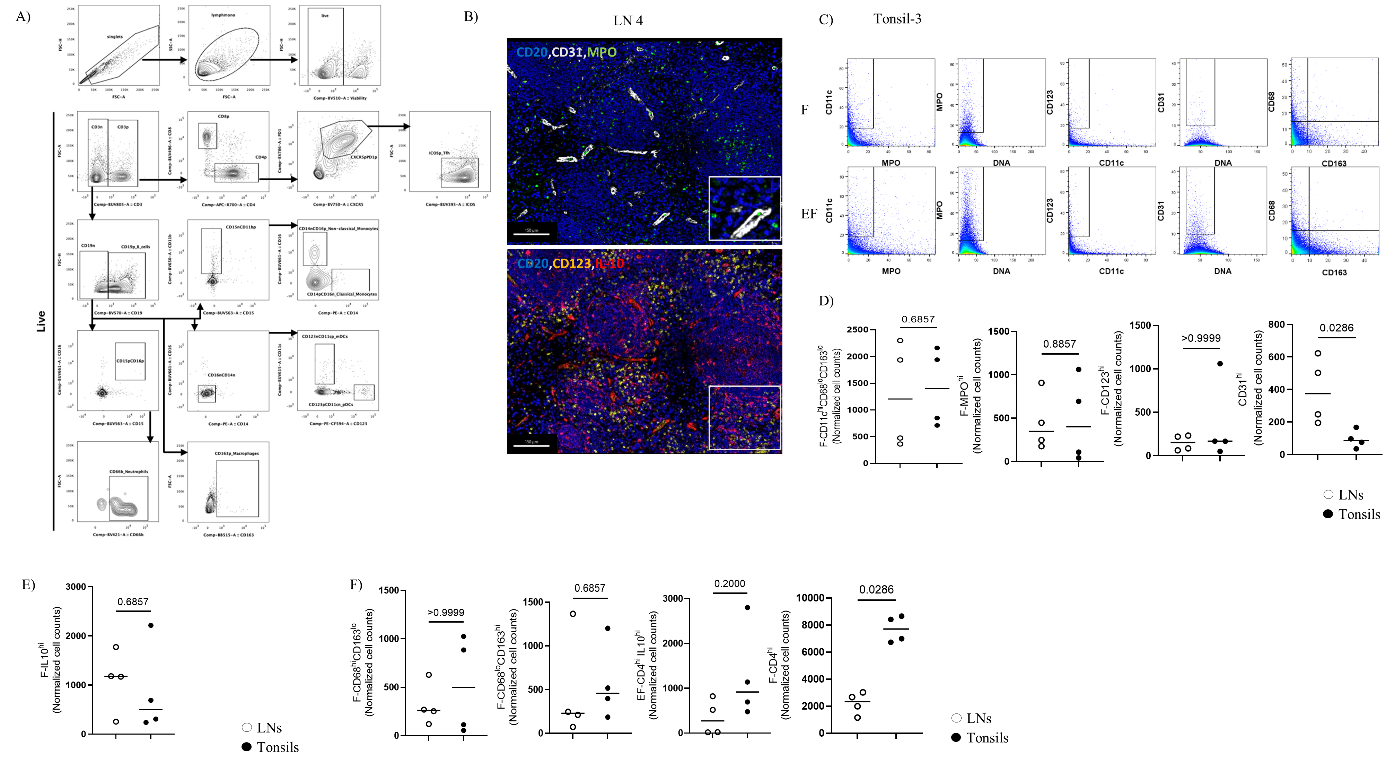


**Suppl. Figure 9. Analyzing innate immune cells and associated cytokines/chemokines. (A)** Flow cytometry gating scheme for the identification of different innate and adaptive immune cell subsets from tonsillar cell suspensions**. (B)** Representative mIF images showing the expression of CD20 (blue), CD31 (white), MPO (green), CD123 (ceramide) and IL10 (red) in a LN (scale bar: 150μm). Inserts show zoomed areas with corresponding biomarkers. **(C)** Histocytometry gating for the identification of innate immunity cell subsets and endothelial cells (CD31^hi^) in F (upper row) and EF (lower row). **(D)** Dot plots showing the normalized cell counts of follicular innate immune cell subsets in LNs (n=4, open circle) and tonsils (n=4, closed circle) for all tissues used. Each circle represents a different donor. **(E)** Dot plot showing the normalized cell counts of bulk follicular IL-10^hi^ cells in all tissues used. Statistical analysis was performed using Mann-Whitney test and significant p values are listed. **(F)** Dot plots showing the normalized cell counts of follicular macrophages, bulk and EF-IL-10^hi^ CD4 cell subsets in LNs (n=4, open circle) and tonsils (n=4, closed circle) for all tissues used. Each circle represents a different donor.
